# Enhancing Near‐Infrared Photoluminescence of Ag_8_GeS_6_ Quantum Dots Through Compositional Fine‐Tuning and ZnS Coating for In Vivo Bioimaging

**DOI:** 10.1002/smll.202411142

**Published:** 2025-05-07

**Authors:** Nurmanita Rismaningsih, Junya Kubo, Masayuki Soto, Kazutaka Akiyoshi, Tatsuya Kameyama, Takahisa Yamamoto, Hiroshi Yukawa, Yoshinobu Baba, Tsukasa Torimoto

**Affiliations:** ^1^ Graduate School of Engineering Nagoya University Chikusa‐ku Nagoya 464–8603 Japan; ^2^ Research Institute for Quantum and Chemical Innovation Institutes of Innovation for Future Society Nagoya University Chikusa‐ku Nagoya 464–8603 Japan; ^3^ Institute for Quantum Life Science National Institutes for Quantum Science and Technology Anagawa 4‐9‐1, Inage‐ku Chiba 263–8555 Japan; ^4^ Department of Quantum Life Science Graduate School of Science Chiba University Chiba 265–8522 Japan

**Keywords:** bioimaging, colloidal Ag_8_GeS_6_ quantum dots, low toxicity, near‐infrared photoluminescence

## Abstract

Quantum dots (QDs) composed of a group I–IV–VI semiconductor, Ag_8_GeS_6_, have been intensively investigated for constructing efficient energy conversion systems. However, their potential for photoluminescence (PL)‐based applications has remained unexplored. Herein, the first successful preparation of Ag_8_GeS_6_ QDs exhibiting near‐infrared (NIR) PL is reported. These Ag_8_GeS_6_ QDs with an average diameter of 4.2–4.6 nm has an almost constant energy gap at 1.48–1.45 eV, even when the Ge/(Ag+Ge) precursor ratio is varied from 0.05 to 0.90. A significant PL peak is observed at 920 nm, the intensity being enlarged with an increase in the Ge/(Ag+Ge) ratio. The use of Ag_8_GeS_6_ QDs prepared with Ge/(Ag+Ge) = 0.82 in the precursors result in a PL quantum yield (QY) of 11%, which is further enhanced to 40% through surface coating with a ZnS shell of 1.0 nm in thickness, with the PL peak wavelength being slightly blue‐shifted to 900 nm. Following surface modification with 3‐mercaptopropionic acid for homogeneous dispersion in aqueous solutions, the Ag_8_GeS_6_@ZnS QDs are utilized as an NIR PL probe for in vivo bioimaging. PL signals are clearly detected from depths of at least 15 mm beneath the back skin of a mouse, demonstrating their deep‐tissue imaging capability.

## Graphical abstract

Fine‐tuning the composition of Ag_8_GeS_6_ quantum dots result in the development of near‐IR photoluminescence. The PL intensity is significantly enhanced by coating with a ZnS shell, enabling 3D near‐IR in vivo bioimaging.

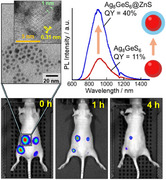

## 1 Introduction

Quantum dots (QDs) with the energy gap in the near‐infrared (NIR) wavelength region have captured significant interest for their wide applicability in various fields, including applications in NIR light‐emitting diodes (LEDs), solar cells, night‐vision devices, fiber‐optic communications, biomedical imaging, and clinical diagnostics.^[^ ^1^, ^2^, ^3^, ^4^, ^5^, ^6^, ^7^, ^8^ ^]^ These QDs exhibit unique optoelectronic properties, such as controllability of the energy gap (*E*_g_), tunable photoluminescence (PL) peak, and high PL quantum yield (QY), originating from the quantum confinement effect. These properties can be precisely controlled by adjusting the size of the QDs.^[^ ^9^, ^10^, ^11^, ^12^ ^]^ In biomedical imaging, QDs are particularly promising due to their superior photostability, tunable emission spectra in the wavelength ranges of the first biological window (700–900 nm) and the second biological window (900–1400 nm), and enhanced biocompatibility when compared to conventional organic fluorophores or proteins.^[^ ^6^, ^13^, ^14^ ^]^ So far, binary QDs composed of II–VI and IV–VI semiconductors such as CdTe,^[^ ^15^, ^16^ ^]^ CdSe,^[^ ^17^, ^18^ ^]^ PbS,^[^ ^19^, ^20^ ^]^ and HgTe^[^ ^21^, ^22^ ^]^ have been most commonly and extensively studied because of their high PL QYs and established synthesis methods. However, their practical use has been severely restricted due to the content of heavy metal elements with high toxicity such as Cd, Pb, and Hg.

To address this critical issue, multinary QDs, such as Ag–In–Ga–Se,^[^ ^23^ ^]^ Ag–In–Te,^[^ ^24^ ^]^ and Zn–Cu–In–Se,^[^ ^25^ ^]^ have attracted significant attention as more environmentally friendly alternatives. Furthermore, since the properties of multinary QDs are dependent on their chemical composition as well as their particle size, such versatility can facilitate the ease of preparing QDs with desired optical characteristics.^[^ ^26^, ^27^ ^]^ For example, Yang et al. reported that the cation exchange of Ag_2_Se QDs with Au^3+^ ions produced alloyed Ag–Au–Se QDs that exhibited an NIR PL peak at 978 nm with PL QY of 65.3% due to the suppression of cation vacancies and/or crystal defects formed by highly mobile Ag^+^ ions in the crystal.^[^ ^28^ ^]^ Pons et al. reported that the long PL lifetime of Zu–Cu–In–Se QDs, 150–300 ns, enabled selective detection of their NIR PL in biological tissues through time‐gated imaging, eliminating the influence of autofluorescence with a faster lifetime, 1−10 ns.^[^ ^25^ ^]^ We have reported that Ag–In–Ga–Se QDs showed a sharp band‐edge emission without a broad defect‐site emission, the peak wavelength being blue‐shifted from 890 to 630 nm with an increase in the Ga^3+^ fraction in QDs.^[^ ^23^ ^]^ Surface coating of Ag–In–Ga–Se QDs with a thin GaS_x_ shell increased the band‐edge PL intensity, with the highest PL QY reaching 38% for the PL peak at 800 nm. Furthermore, alloying S^2−^ into these QDs also enabled a blue shift of the emission peak from 790 to 580 nm with an increase in the S fraction in the resulting Ag–In–Ga–S–Se QDs.^[^ ^29^ ^]^ After coating with a GaS_x_ shell, Ag–In–Ga–S–Se QDs with the band‐edge PL peak at 790 nm were encapsulated into liposomes, and the resulting QD‐liposome composites with PL QY of 19% were successfully applied to in vivo bioimaging.^[^ ^29^ ^]^

Recently, I–IV–VI semiconductors, such as Cu_2_SnS_3_, Ag_8_SnS_6_, Ag_8_GeS_6_, and Ag_8_(Ge,Sn)(S,Se)_6_, have attracted much attention for the development of highly efficient solar cells and thermoelectrics owing to their controllable *E*_g_ in the NIR region by the alloy composition.^[^ ^30^, ^31^, ^32^, ^33^, ^34^ ^]^ QDs consisting of such semiconductors were also investigated to develop cost‐effective devices via wet chemical processing. Among them, argyrodite Ag_8_GeS_6_ QDs seem to be an ideal candidate as a light absorber for both photocatalytic and photovoltaic devices^[^ ^30^, ^33^, ^35^, ^36^, ^37^ ^]^ because they are composed of less toxic and earth‐abundant elements and their bulk band gap, 1.48 eV,^[^ ^38^ ^]^ is sufficiently narrow for effective utilization of solar light. Polymer solar cells were fabricated by Li et al. with the use of Ag_8_GeS_6_ QDs as a light‐absorbing material.^[^ ^36^ ^]^ Zhou et al. reported the preparation of Ag_8_(Ge,Sn)(S,Se)_6_ QDs for application to NIR photovoltaics, in which the *E*_g_ of the QDs was increased from 0.88 to 1.45 eV with a decrease in the Se fraction.^[^ ^30^ ^]^ Van Embden et al. found that phase segregation occurred in Ag_8_GeS_6_ nanocrystals due to the high ionic mobility of Ag^+^ ions, resulting in the formation of a Janus‐type Ag–Ag_8_GeS_6_ heterostructure in single nanocrystals.^[^ ^37^ ^]^ Recently, the synthesis of bulk α’‐Ag_8_GeS_6_ crystals showing emission exclusively at very low temperatures, below 130 K, was reported.^[^ ^38^ ^]^ The PL spectra of those crystals featured a sharp peak at 1.57 eV (790 nm), attributed to band‐edge emission, and a broad peak at 1.35 eV (919 nm), originating from defect sites. However, there has been no report on photoluminescence properties of Ag_8_GeS_6_ QDs at room temperature.

Here, we report solution‐phase synthesis of Ag_8_GeS_6_ QDs emitting an NIR PL for the first time and their potential ability as a luminescent probe for in vivo bioimaging. A broad PL peak arising from defect sites was observed at 900 nm by precisely controlling the Ge/Ag ratio in the precursor. The PL intensity was remarkably enhanced by surface coating with a ZnS shell of 1.0 nm in thickness, accompanied by a slight blue shift of the PL peak to 900 nm, in which the PL QY was 40% and the average lifetime of PL was as long as 486 ns. Surface modification with a hydrophilic ligand enabled uniform dispersion of Ag_8_GeS_6_@ZnS QDs in aqueous solutions, and the QDs showed very low cytotoxicity and were applied to in vivo bioimaging in the NIR wavelength range.

## 2 Experimental Methods

### 2.1 Materials

Germanium (IV) oxide was purchased from Kojundo Chemical Laboratory Co., Ltd., and silver (I) diethyldithiocarbamate (AgDDTC) was obtained from Sigma‐Aldrich. Glycolic acid and zinc (II) stearate were obtained from FUJIFILM Wako Pure Chemical Corporation. Thiourea and thioacetamide were purchased from Kishida Reagents Chemicals. These chemicals were used as precursors for the preparation of QDs. Oleylamine (OLA, Sigma–Aldrich) and 1‐dodecanethiol (DDT, FUJIFILM Wako Pure Chemical Corporation) were used as solvents for the synthesis of QDs. For the ligand exchange, tetramethylammonium hydroxide (TMAOH, 25 wt% in methanol, Sigma–Aldrich) and 3‐mercaptopropionic acid (MPA, Tokyo Chemical Industry Co., Ltd.) were used. Dulbecco's modified Eagle's medium (DMEM)/F12 was obtained from Thermo Fisher Scientific K.K. (Tokyo, Japan), and fetal bovine serum (FBS) was obtained from Trace Scientific Ltd. (Melbourne, Australia). Other reagents were obtained from Kishida Reagents Chemicals. To remove water, oxygen, and carbon dioxide dissolved, OLA was heated at 60 °C for 1 h under vacuum. Other chemicals were used as received without further purification. Aqueous solutions were prepared using water purified by a Sartorius Arium system.

HeLa cells (Cell line, Human cervical cancer, Female) and Swiss 3T3 cells (Cell line, Mouse fibroblast, Embryo) were purchased from RIKEN BRC (RCB0007, RCB1642) in Japan. The cells were cultured according to the protocols shown by RIKEN BRC. Briefly, HeLa cells (RCB0007) and Swiss 3T3 cells were incubated with Dulbecco's modified Eagle's medium (DMEM; Gibco 11965‐032, Thermo Fisher Scientific, Japan) containing 10% fetal bovine serum (FBS; S1810, Biowest, France) and 100 U cm^−3^ penicilin/streptomycin (Gibco 15140122, Thermo Fisher Scientific, Japan) at 37 °C with 5% CO_2_.

Female BALB/c mice (6 weeks old) were purchased from SLC Japan (Hamamatsu, Japan) for use in in vivo toxicity testing. The mice were housed in a controlled environment (12‐h light/dark cycles at 22 °C) with free access to water and a standard chow diet before sacrifice. All conditions and handling of animals in this study were conducted with protocols approved by the Nagoya University Committee on Animal Use and Care (No. M240295‐002).

### 2.2 Synthesis of Ag_8_GeS_6_ QDs by a Heating‐Up Method

As a Ge precursor with high stability against air and moisture, diaquabis(glycolato‐O,O’)germanium(IV) (Ge(gly)_2_(H_2_O)_2_) was prepared from germanium (IV) oxide and glycolic acid according to the procedure reported by Chesman et al.^[^ ^35^ ^]^ Ag_8_GeS_6_ QDs were synthesized using a heating‐up method similar to that in our previous work^[^ ^29^ ^]^ with slight modifications. Powders of AgDDTC and Ge(gly)_2_(H_2_O)_2_ were used as metal ion precursors, while thiourea served as an S^2−^ precursor. A typical synthesis strategy is as follows. The metal ion precursors and the S^2−^ precursor were put in a test tube with a mixed solvent of 2.75 cm^3^ OLA and 0.25 cm^3^ DDT. To examine how the Ge fraction affects the optical properties of the synthesized QDs, we systematically varied the Ge content in the metal precursors. The Ge fraction, defined as Ge/(Ag+Ge), was varied from 0.05 to 0.90. During this process, the amount of AgDDTC was kept constant at 0.025 mmol. Additionally, the total positive charge of the metal cations (Ag^+^+Ge^4+^) was maintained in a 1:1 ratio with the negative charge from sulfide ions (S^2−^), which were generated through thiourea decomposition. The reaction solution was degassed under vacuum and then heat‐treated at 150 °C for 20 min with vigorous stirring under an N_2_ atmosphere. The resulting dark‐brown‐colored suspension was cooled to room temperature and was then subjected to centrifugation at 4000 rpm for 5 min to remove large precipitates. Then 3 cm^3^ of methanol was added to the supernatant to precipitate Ag_8_GeS_6_ QDs. After the precipitates had been isolated by centrifugation, they were washed several times with methanol and ethanol and then dissolved in 1.0 cm^3^ of chloroform for further experiments.

### 2.3 Surface Coating of Ag_8_GeS_6_ QDs with a ZnS Shell by a Heating‐Up Method

**Figure 1:**
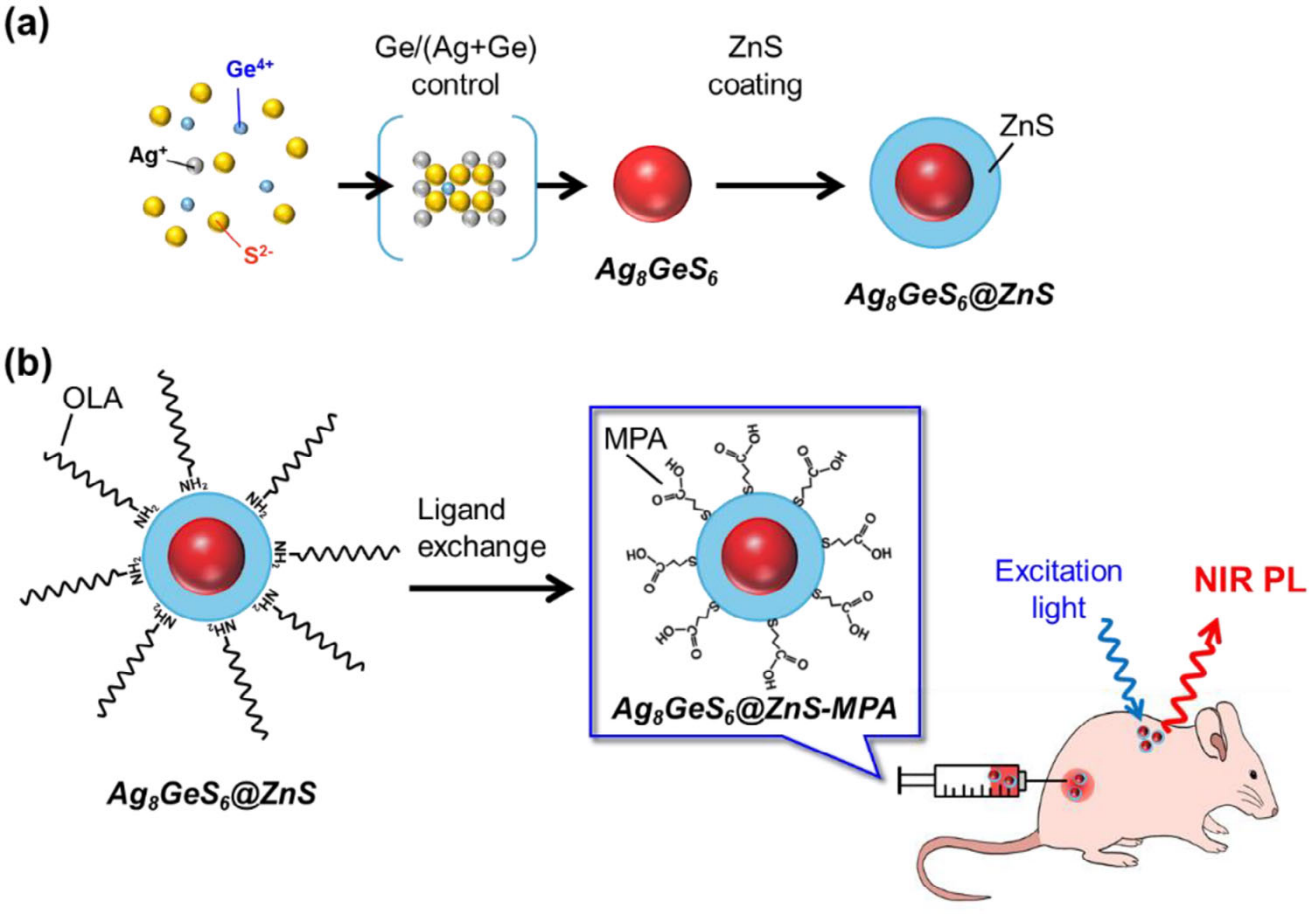
Schematic illustration of in vivo bioimaging with NIR‐luminescent Ag_8_GeS_6_ QDs. a) Preparation of Ag_8_GeS_6_ QDs with various Ge/(Ag+Ge) ratios in the precursors and subsequent surface coating with a ZnS shell. b) NIR in vivo imaging with use of MPA‐modified Ag_8_GeS_6_@ZnS core‐shell QDs.

First, 0.011 mmol of zinc stearate and 0.011 mmol of thioacetamide were put into a test tube containing 1.0 nmol(QDs) of prepared Ag_8_GeS_6_ QDs. After the addition of OLA (3.0 cm^3^) as a solvent, the suspension was heated at 200 °C for 15 min with vigorous stirring under an N_2_ atmosphere. The thus‐obtained suspension was cooled to room temperature and was then subjected to centrifugation at 4000 rpm for 5 min to remove large aggregates. By adding methanol (3 cm^3^) to the supernatant, target QDs with a Ag_8_GeS_6_ core‐ZnS shell structure, denoted as Ag_8_GeS_6_@ZnS (**Figure** **1**a), were isolated, followed by washing several times with methanol and ethanol. The obtained wet precipitates were dissolved in 1.0 cm^3^ of chloroform.

### 2.4 Controlling the ZnS Shell Thickness Deposited on Ag_8_GeS_6_ Cores by Dropwise Addition of a Zn Precursor

To investigate the influence of ZnS shell thickness on the optical properties of Ag_8_GeS_6_ QDs, we increased the shell thickness on Ag_8_GeS_6_ cores by dropwise addition of a Zn precursor solution. In a test tube containing 1.0 nmol(QDs) of Ag_8_GeS_6_ QDs, 0.011 mmol of thioacetamide and 1.0 cm^3^ of OLA were added. Separately, in another test tube, 0.011 mmol of zinc stearate was dispersed in 3.0 cm^3^ of OLA (Zn solution). After being degassed, the solution containing Ag_8_GeS_6_ QDs, thioacetamide, and OLA was subjected to heating at 200 °C. To the solution, 3.0 cm^3^ of Zn solution was added dropwise, with the injection speed being varied from 0.075 to 0.016 cm^3^/min. After the solution had been allowed to cool to room temperature, target Ag_8_GeS_6_@ZnS QDs were isolated and purified in a manner similar to that described in 2.3. The obtained QDs were dissolved in 1.0 cm^3^ of chloroform.

### 2.5 Uniform Dispersion of Ag_8_GeS_6_@ZnS QDs in Aqueous Solutions via Ligand Exchange

Since Ag_8_GeS_6_@ZnS QDs were synthesized with the use of OLA as a surface ligand, they had a hydrophobic organic layer on their particle surface, resulting in poor dispersibility in aqueous solutions. Phase transfer of the QDs into aqueous solutions from organic solutions was achieved by replacing the initial surface ligand of hydrophobic OLA with hydrophilic MPA (Figure 1b), as follows. To 1.0 cm^3^ of a chloroform solution containing as‐prepared Ag_8_GeS_6_@ZnS (0.50 µmol(QDs) dm^−3^), a mixed solution of 0.20 cm^3^ of TMAOH methanol solution (25 wt%), 0.80 cm^3^ of ethanol, and 0.10 cm^3^ of MPA was added. The mixture was vigorously stirred at 70 °C for 3 h under an N_2_ atmosphere to facilitate the ligand exchange reaction. After the solution had been left standing for a few minutes at room temperature, 3 cm^3^ of acetone was added, followed by centrifugation at 4000 rpm for 5 min. The obtained precipitates were washed with acetone. Thus‐obtained QDs denoted as Ag_8_GeS_6_@ZnS‐MPA were re‐dispersed in pure water for further use.

### 2.6 Characterization of Quantum Dots

Specimens for transmission electron microscopy (TEM) measurements were prepared by spreading a QD chloroform solution onto a Cu TEM grid coated with a layer of amorphous carbon (Okenshoji Co., Ltd, ELS‐C10 STEM Cu100P grid), followed by drying under vacuum. TEM images were captured using a Hitachi H‐7650 TEM at an operating voltage of 100 kV, covering a wide area, and the images were used to examine the morphology and size distribution of QDs. A Cs‐corrected HR‐STEM (JEOL Co. Ltd, ARM‐200F) with an acceleration voltage of 200 kV was used to obtain high‐resolution images of high‐angle annular dark‐field scanning transmission electron microscopy (HAADF‐STEM). Nanoscale energy‐dispersive X‐ray spectroscopy (EDS) analysis of individual QDs was carried out during the HAADF‐STEM measurements. In contrast, the chemical composition of QD ensembles was evaluated by an EDS analyzer (Horiba, Emax Energy EX‐250) or an X‐ray fluorescence spectrometer (Rigaku, NEX CG). X‐ray photoelectron spectroscopy (XPS) was conducted to investigate the surface chemical environment of the elements present in the QDs using a PHI Quantes (ULVAC‐PHI) with a conventional monochromatic Al Kα irradiation at 1486.6 eV. A Rigaku SmartLab‐3K X‐ray diffractometer with Cu Kα radiation was used to measure powder X‐ray diffraction (XRD) patterns of QDs. The average hydrodynamic diameter of ligand‐exchanged QDs was evaluated by dynamic light scattering measurements (DLS; Otsuka Electronics FDLS‐3000).

An Agilent 8453A diode array spectrophotometer was used to obtain absorption spectra. PL spectra were measured at room temperature using photonic multichannel analyzers (Hamamatsu, PMA‐12, C10027−02 for the 350−1100 nm range and C10028−01 for the 900−1650 nm range), and the PL spectra were displayed by merging the obtained spectra at 1000 nm if required. The wavelength of excitation light was 600 nm. The PL QY was determined using indocyanine green as a standard reference for near‐infrared photoluminescence. The relative PL QY of QDs with 600 nm excitation light was estimated by the following Equation (1):

(1) $$\Phi_{\mathrm{QD}}=\Phi_{\mathrm{st}}\times \left(\frac{FA_{\mathrm{QD}}}{FA_{\mathrm{st}}}\right)\times \left(\frac{A_{\mathrm{st}}}{A_{\mathrm{QD}}}\right)\times \left(\frac{I_{\mathrm{ex},\;\mathrm{st}}}{I_{\mathrm{ex},\;\mathrm{QD}}}\right)\times \left(\frac{{n_{\mathrm{QD}}}^{2}}{{n_{\mathrm{st}}}^{2}}\right)$$

where *Φ* is the quantum yield of samples, *FA* is the measured integrated PL intensity, *A* is the absorbance at the excitation wavelength (typically 0.1), *I*_ex_ is the intensity of excitation light at the excitation wavelength, *n* is the refractive index of the solvent used, and the subscripts QD and ST refer to parameters of the QD solution and the standard reference, respectively. An ethanol solution (n = 1.3618) of indocyanine green (ICG; Φ = 13.2%^[^ ^39^ ^]^), which is an organic fluorescent dye that emits near‐infrared light, was used as a standard sample, the excitation being performed at 700 nm. PL decay profiles were acquired by using a time‐correlated single‐photon counting apparatus (Hamamatsu, Quantaurus‐Tau).

By using photoelectron yield spectroscopy in air (PYSA) (Riken Keiki, AC‐2), we investigated the ionization energy of QDs. Samples were prepared by spin‐coating an Ag_8_GeS_6_ QD chloroform solution onto an indium tin oxide (ITO) substrate, followed by drying under an N_2_ flow. In general, a linear relationship is observed between the *n*th power of photoelectron emission yield and the excitation photon energy for metals and semiconductors, in which the *n*th value has been empirically chosen between 0.3 and 1.0.^[^ ^40^, ^41^ ^]^ In this study, we plotted the square root (*n* = 0.5, the most common *n* value for PYSA measurements) of photoelectron emission yield against the excitation photon energy. The threshold photon energy for photoelectron emission was determined from the intercept between the linear portion of the PYSA spectrum increase and the baseline, representing the ionization energy that is assumed to correspond to the energy of the valence band maximum of a semiconductor.

### 2.7 In Vivo Bioimaging Using Ag_8_GeS_6_@ZnS‐MPA QDs

Figure 1b shows a schematic illustration of in vivo bioimaging. NIR‐photoluminescent QDs of Ag_8_GeS_6_@ZnS‐MPA, with a shell thickness of 1.0 nm and uniformly dispersed in water, were used for in vivo bioimaging via both subcutaneous injection and intravenous injection. The step‐by‐step guide of the procedures was similar to that described in a previous publication of our group.^[^ ^29^ ^]^ BALB/c mice were used for in vivo bioimaging.

For subcutaneous PL imaging, the QDs were diluted with phosphate‐buffered saline (PBS) (pH 7.4) to obtain QD concentrations of 0.10–0.80 µmol(QDs) dm^−3^. A 50‐mm^3^ portion of each dispersion was injected subcutaneously into the back of a mouse under anesthesia. For the intravenous injection, the QDs were diluted with phosphate‐buffered saline (PBS) (pH 7.4) to a concentration of 0.80 µmol(QDs) dm^−3^. A 0.20‐cm^3^ portion of the dispersion was injected intravenously into a mouse via the tail vein. PL signals were detected with an in vivo PL imaging system (IVIS Spectrum CT) at various time points after injection. The imaging settings were as follows: an excitation filter at 640 nm and an emission filter at 840 nm were used for subcutaneous imaging, while an excitation filter at 710 nm and an emission filter at 840 nm were used for intravenous injection. To identify the organs emitting PL, an organ atlas was applied to the fluorescence images using Living Image software.^[^ ^42^ ^]^ The experimental procedures involving animal subjects were approved by the Nagoya University Committee on Animal Use and Care (No. 20 242).

### 2.8 Durability of the PL Intensity of Ag_8_GeS_6_@ZnS‐MPA QDs Under Various Conditions

The PL stability of Ag_8_GeS_6_@ZnS‐MPA QDs in water was evaluated by measuring their PL intensity under various conditions. The QDs were dissolved in water to achieve an absorbance of 0.1 at 600 nm. A prepared solution was stored in the dark at 4 °C under an N_2_ atmosphere and then PL spectra were measured intermittently for several months. Furthermore, another solution was exposed to irradiation with monochromatic light at 700 nm with an intensity of 4.5 mW cm^−2^ at room temperature. The PL spectra were measured at various irradiation times.

The PL stability of Ag_8_GeS_6_@ZnS‐MPA QDs in different biological media was further investigated. By dispersing the QDs in water to achieve an absorbance of 0.2 at 600 nm, the resulting QD solutions were mixed with equal volumes of four different types of biological media: DMEM, DMEM supplemented with 10% of FBS (DMEM‐FBS), FBS, and PBS. The obtained mixtures were stored in the dark at 4 °C under an N_2_ atmosphere. The PL intensity of each mixture was monitored at regular intervals to assess the impact of different biological media on PL properties of the QDs.

### 2.9 In Vitro Cytotoxicity Testing of Ag_8_GeS_6_@ZnS QDs

In vitro cytotoxicity was evaluated using protocols similar to those used in previous studies with minor modifications.^[^ ^29^, ^43^, ^44^ ^]^ HeLa cells and Swiss 3T3 cells (1 × 10⁴ cells/well) were seeded in 96‐well plates (BD Biosciences) with 0.10 cm^3^ of a culture medium consisting of DMEM, 10% FBS and 100 U/cm^3^ penicillin/streptomycin. The cells were incubated for 24 h at 37 °C in a CO_2_ incubator. An Ag_8_GeS_6_@ZnS aqueous solution (0.60 µmol (QDs) dm^−3^) was diluted with FBS‐free DMEM medium and applied to the cells at various concentrations. After 24 h of incubation, the cells were washed with FBS‐free DMEM medium, and 0.10 cm^3^ of FBS‐free DMEM medium was added to each well. For cytotoxicity assessment, viable cells were quantified using the Cell Counting Kit‐8 (CCK‐8; DOJINDO Laboratories, Japan). CCK‐8 solution (0.010 cm^3^) was added to each well, and the reaction was allowed to proceed for up to 2 h. The absorbance of the sample at 450 nm was measured against a background control using a microplate reader (Infinite 200 PRO; Tecan Japan Co., Ltd., Kawasaki, Japan).

### 2.10 In Vivo Toxicity Testing of Ag_8_GeS_6_@ZnS QDs

Female BALB/c mice were used to evaluate the in vivo toxicity of Ag_8_GeS_6_@ZnS QDs. A 6.0 nmol(QDs) dm^−3^ QD solution was prepared by diluting a 0.60 µmol(QDs) dm^−3^ Ag_8_GeS_6_@ZnS QDs stock solution with PBS. Mice were divided into two groups: an Ag_8_GeS_6_@ZnS QD group (3 mice) and a PBS control group (1 mouse), each receiving a 0.10 cm^3^ subcutaneous injection. Blood samples were collected from the heart under isoflurane anesthesia at 1, 7, and 14 days after the QD injection. Serum was analyzed biochemically by Oriental Yeast Co., Ltd. Major organs (liver, spleen, kidneys, intestine, lungs, and heart) were harvested and examined using an IVIS Spectrum CT system (excitation filter: 640 nm, emission filter: 840 nm) to assess biodistribution.

### 2.11 Statistical Analysis

Data are presented as individual values or as means ± standard deviation (SD), where applicable. Measurements were conducted under consistent experimental conditions, and variations in data were within an acceptable range for reproducibility. Statistical significance was determined using Student's t‐test.

## 3 Results and Discussion

### 3.1 Composition‐Dependent PL Properties of Ag_8_GeS_6_ QDs

**Figure 2:**
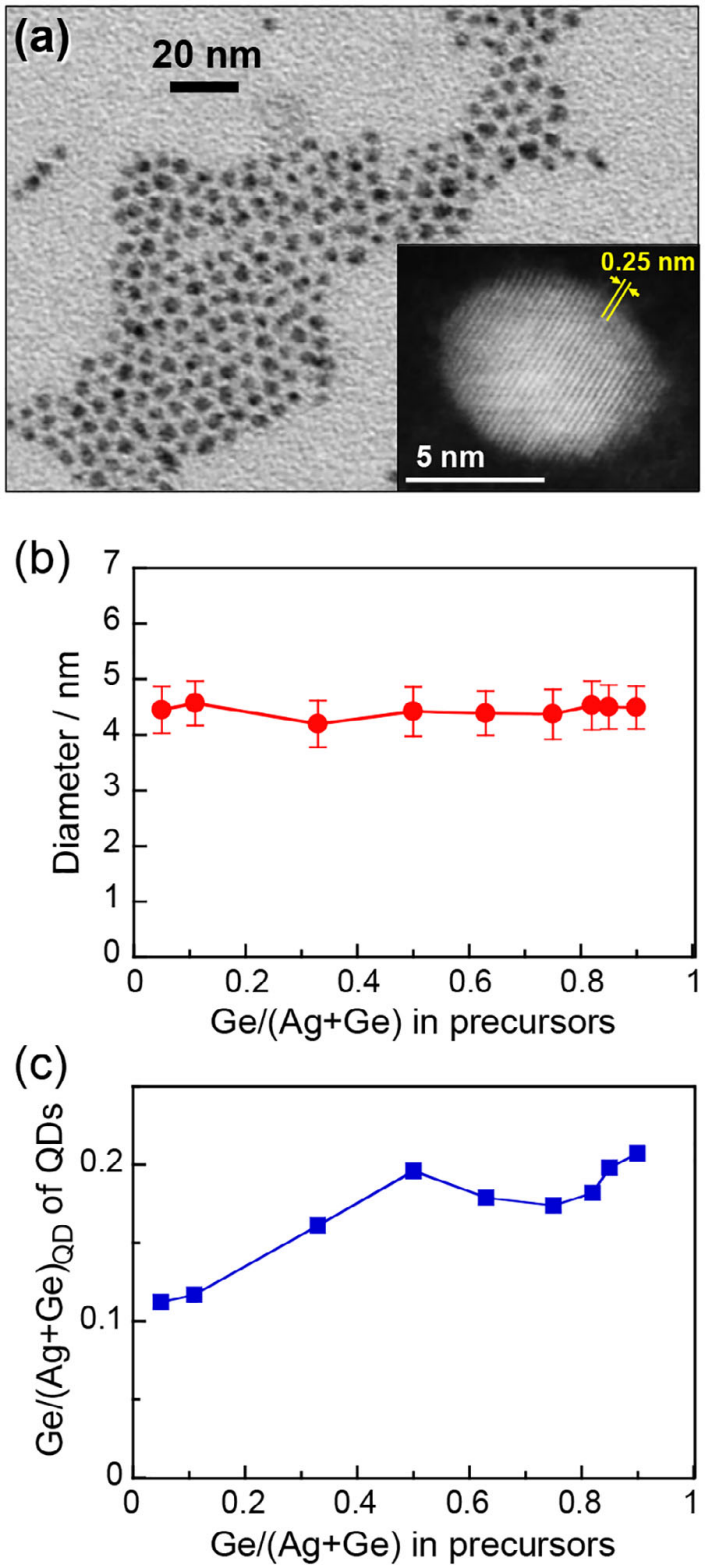
(a) A representative TEM image of Ag_8_GeS_6_ QDs prepared with Ge/(Ag+Ge) = 0.82. (Inset) A high‐resolution HAADF‐STEM image of an individual QD. (b) Dependence of the average size of Ag_8_GeS_6_ QDs on the Ge/(Ag+Ge) ratio in the precursors. The error bars represent the standard deviation. (c) Relationship between the experimentally obtained Ge/(Ag+Ge)_QD_ ratio of QDs and that used in the precursors.

Ag_8_GeS_6_ QDs were prepared through a heating‐up method, in which a thermal reaction occurred between metal precursors and thiourea in an OLA‐DDT mixture solution. Thus‐obtained QDs were spherical or polygonal particles with a relatively narrow size distribution as shown in **Figures** **2**a,b and S1 (Supporting Information). The average diameter (*d*_av_) of the particles was almost constant at 4.2–4.6 nm with a standard deviation of ca. 10% of *d*_av_, regardless of the Ge/(Ag+Ge) ratio in the precursors. However, the chemical composition of QDs varied, as shown in Table S1 (Supporting Information), depending on the precursor ratio. The Ge fractions in total metals of Ag_8_GeS_6_ QDs, denoted as Ge/(Ag+Ge)_QD_, were experimentally obtained as shown in Figure 2c. QDs prepared with Ge/(Ag+Ge) = 0.050 in the precursors had a stoichiometric composition for the Ag_8_GeS_6_ crystal, which was Ge/(Ag+Ge)_QD_ = 0.11. In contrast, with an increase in the Ge/(Ag+Ge) ratio in the precursors from 0.11 to 0.90, the Ge fraction in the obtained QDs exhibited a tendency to increase from Ge/(Ag+Ge)_QD_ = 0.12 to 0.21, suggesting the formation of a non‐stoichiometric Ge‐rich Ag_8_GeS_6_ crystal. A high‐resolution HAADF‐STEM image of Ag_8_GeS_6_ QDs prepared with Ge/(Ge+Ag) = 0.82 in the precursors is shown in the inset of Figure 2a. A continuous lattice fringe with interplanar spacing of 0.25 nm, assignable to the (6 0 0) plane of orthorhombic Ag_8_GeS_6_ (0.253 nm), was observed throughout a particle in the high‐resolution HAADF‐STEM image. The uniform lattice structure across the entire QD, along with the absence of observable grain boundaries, strongly suggests that the obtained QDs are highly crystalline. Notably, no amorphous layer was detected on the surface of Ag_8_GeS_6_ QDs in the HAADF‐STEM image, confirming their high purity and crystallinity.

**Figure 3:**
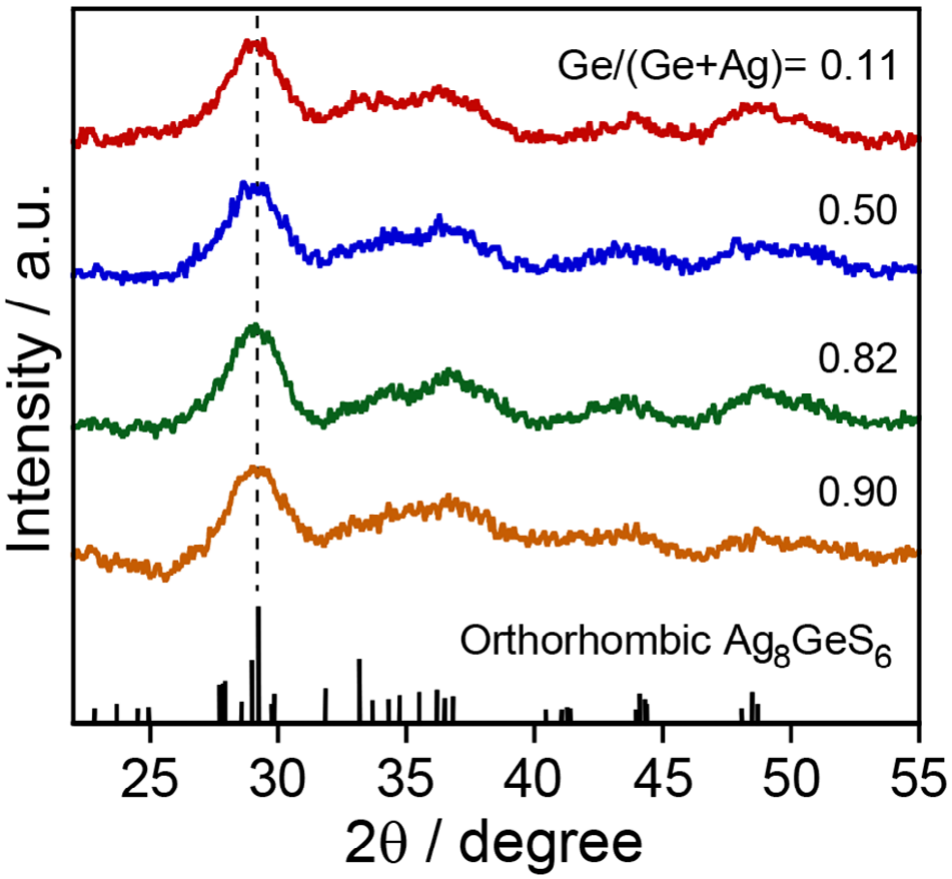
XRD patterns of Ag_8_GeS_6_ QDs prepared with different Ge/(Ag+Ge) ratios. The standard diffraction pattern of orthorhombic Ag_8_GeS_6_ (PDF card# 00‐044‐1416) is shown as a reference.

**Figure** **3** shows XRD patterns of Ag_8_GeS_6_ QDs obtained with different Ge/(Ag+Ge) ratios in the precursors. Broad diffraction peaks were observed due to their size of several nanometers. Each diffraction pattern agreed well with orthorhombic Ag_8_GeS_6_ known as an argyrodite structure, regardless of the ratio of metal precursors: The highest diffraction peak at the 2*θ* angle of ca. 29° corresponds to the (0 2 2) plane of orthorhombic Ag_8_GeS_6_, 29.23°. No significant peak shift was observed when the Ge fraction in Ag_8_GeS_6_ QDs was increased from 0.11 to 0.90. Thus, these results indicate that the present synthetic method provided a series of Ag_8_GeS_6_ QDs of a constant size but different compositions, which are suitable for investigating the dependence of the optical properties of Ag_8_GeS_6_ QDs on the chemical composition.

**Figure 4:**
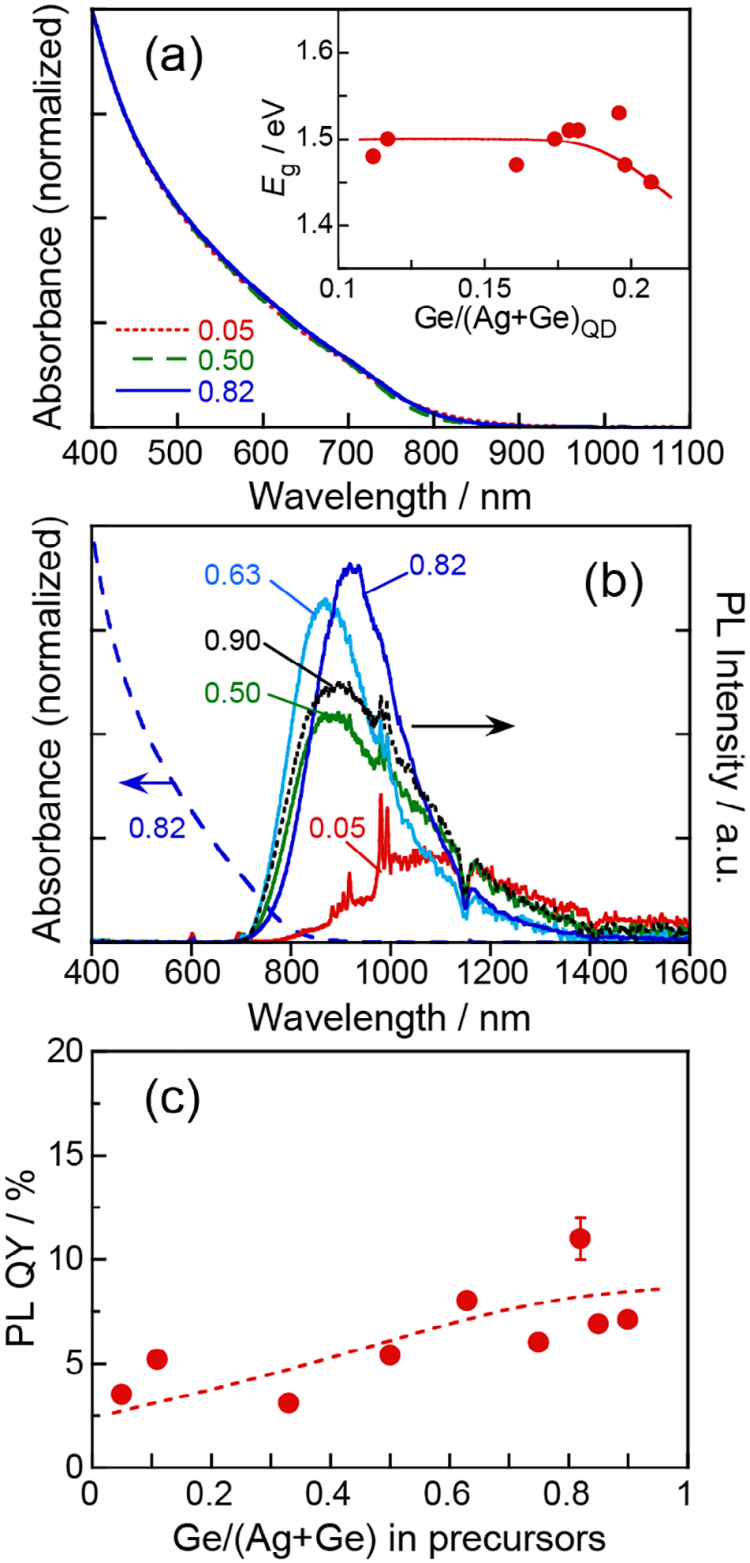
Absorption a) and PL spectra b) of Ag_8_GeS_6_ QDs prepared with different Ge/(Ag+Ge) ratios. The numbers represent the ratios of Ge/(Ag+Ge) in the precursors. The inset panel shows the dependence of *E_g_* of QDs on the Ge/(Ag+Ge)_QD_ ratio of the QDs. Spike signals observed ≈1000 nm in panel b were attributed to stray light from the excitation light source. c) PL QYs of Ag_8_GeS_6_ QDs prepared with different Ge/(Ag+Ge) ratios.

The absorption spectra of Ag_8_GeS_6_ QDs are shown in **Figure** **4**a. Similar broad structureless spectra were observed, regardless of the Ge fraction in precursor, in which the absorption onset appeared at ≈850 nm. From Tauc plots of the absorption spectra (Figure S2, Supporting Information), we determined the *E*_g_ value of the QDs. The estimated *E*_g_ value decreased from 1.48 eV to 1.45 eV with an increase of the Ge/(Ag+Ge) ratio in precursors from 0.050 to 0.90, being slightly larger or equal to that of bulk Ag_8_GeS_6_, 1.45 eV: Considering the change in the chemical composition of the obtained QDs, the *E*_g_ value tended to be slightly lower with an increase in the Ge fraction in the QDs (Figure 4a inset), suggesting the formation of intragap states originating from excess Ge^4+^ ions in the crystal, such as Ag^+^ vacancies, Ge^4+^ interstitials, and antisites of Ge^4+^ on Ag^+^ sites, in Ge‐rich Ag_8_GeS_6_ QDs.

We evaluated the electronic energy structure of Ag_8_GeS_6_ QDs by measuring the ionization energy with photoemission yield spectroscopy in air (PYSA). As shown in FigureS3 (Supporting Information), the ionization energy of QDs tended to decrease from 5.53 eV to 5.44 eV with an increase in the Ge/(Ag+Ge) ratio in the precursors from 0.05 to 0.90. The energy level of the valence band maximum (VBM) was estimated as the value with the opposite sign of the ionization energy of semiconductors. Subsequently, the level of the conduction band minimum (CBM) was determined by subtracting the *E*_g_ value from the VBM. Figure S4 (Supporting Information) shows the dependence of VBM and CBM levels on the experimentally obtained Ge fraction of Ag_8_GeS_6_ QDs. Both the levels of VBM and CBM were linearly shifted to higher levels with an increase in the Ge fraction of QDs: The VBM was moved from –5.53 eV for the stoichiometric Ag_8_GeS_6_ QDs (Ge/(Ag+Ge)_QD_ = 0.11) to –5.35 eV for Ge‐rich QDs with Ge/(Ag+Ge)_QD_ = 0.21, while the CBM was varied from –4.05 eV for the stoichiometric QDs to –3.90 eV for the Ge‐rich QDs. It has been reported from theoretical calculations based on density functional theory for Ag_8_GeS_6_^[^ ^38^, ^45^ ^]^ that the VBM was composed of S 3*p* and Ag 4*d* orbitals and that the CBM was mainly formed by mixing *p* orbitals of S and *s,p* orbitals of Ag and Ge. This can explain the upward shift of the CBM level of Ag_8_GeS_6_ QDs with an increase in the Ge fraction. However, the change in the VBM level of Ge‐rich Ag_8_GeS_6_ QDs in comparison to stoichiometric QDs was not explained by the orbitals of elements theoretically expected to form the VBM. If excess Ge^4+^ ions incorporated in the Ag_8_GeS_6_ crystal can form intragap states above the valence band, the VBM level of QDs is apparently shifted to a higher level with an increase in the Ge fraction of QDs. This is probably true of the present case.

Figure 4b shows photoluminescence (PL) spectra of Ag_8_GeS_6_ QDs with different compositions. A broad PL peak was observed in the range of 700–1400 nm for each type of QDs, in which the full width at half maximum intensity (FWHM) of the PL peak was 200–300 nm. The peak wavelengths were 860–920 nm near the absorption onset wavelength, except for the QDs prepared with Ge/(Ag+Ge) = 0.05 showing the PL peak top at 1090 nm. Considering the significantly large peak width, the PL of the present QDs was not assigned to the band‐edge emission but originated from the defect sites. Furthermore, the PL intensity was dependent on the Ge/(Ag+Ge) ratio in the precursors. Figure 4c shows the dependence of PL QY on the Ge/(Ag+Ge) ratio in the precursors. The PL QY tended to be larger with an increase in the Ge/(Ag+Ge) ratio. The highest PL QY was 11% for QDs prepared with Ge/(Ag+Ge) = 0.82 showing a PL peak at 920 nm. The observed increase in PL QY with higher Ge/(Ag+Ge) ratios suggests that Ge‐rich compositions are more likely to promote defect‐related PL emission.

The PL excitation (PLE) spectrum of Ag_8_GeS_6_ QDs (Figure S5, Supporting Information) closely matched the corresponding absorption spectrum for wavelengths below 750 nm, indicating that the observed PL was induced by the photoexcitation of Ag_8_GeS_6_ QDs even with NIR lights in the range of 700–750 nm. It should be noted that the PLE spectrum beyond 750 nm could not be accurately measured due to the overlapping between the excitation light and the PL from QDs, though Ag_8_GeS_6_ QDs exhibited light absorption from 750 to ca. 850 nm in the first biological window (Figure 4a). The intensity of the PL peak also varied with an increase in the synthesis temperature. With an increase in the reaction temperature from 150 °C to 220 °C, the PL intensity of the obtained QDs significantly decreased to less than one‐tenth (Figure S6, Supporting Information), while the absorption spectra were almost constant. The average QD sizes were also increased from *d*_av_ = 4.3 nm at 150 °C to 5.9 nm at 220 °C. These results suggested that the amount of radiative defect sites in a QD crystal was controlled by the reaction temperature and/or the QD size. Consequently, for the subsequent experiments, we selected the optimal synthesis temperature of 150 °C for the preparation of Ag_8_GeS_6_ QDs.

### 3.2 Enhancing PL Properties of Ag_8_GeS_6_ QDs with a ZnS Shell Coating

Quantum dots have defect sites on their surface, which cause the formation of non‐radiative recombination sites and intragap states. One useful strategy for removing such sites is surface coating QDs with different semiconductors that have wider bandgaps, creating core–shell particles with a type‐I heterostructure. This approach eliminates defect sites and harmful dangling bonds on the core surface and confines photogenerated charge carriers within the core, resulting in improvement of the PL properties and photostability of QD cores. Zinc sulfide (ZnS) with *E*_g_ = 3.7 eV is frequently used as a shell material for binary QDs such as CdS^[^ ^46^ ^]^ and CdSe^[^ ^47^ ^]^ and multinary QDs such as AgInS_2_^[^ ^48^, ^49^ ^]^ and CuInS_2_^[^ ^50^, ^51^ ^]^ due to the similarity of crystal structure and a small lattice mismatch at the interface between the core and shell. Thus, we evaluated the change in the optical properties of Ag_8_GeS_6_ QDs by coating ZnS shells with different thicknesses.

**Figure 5:**
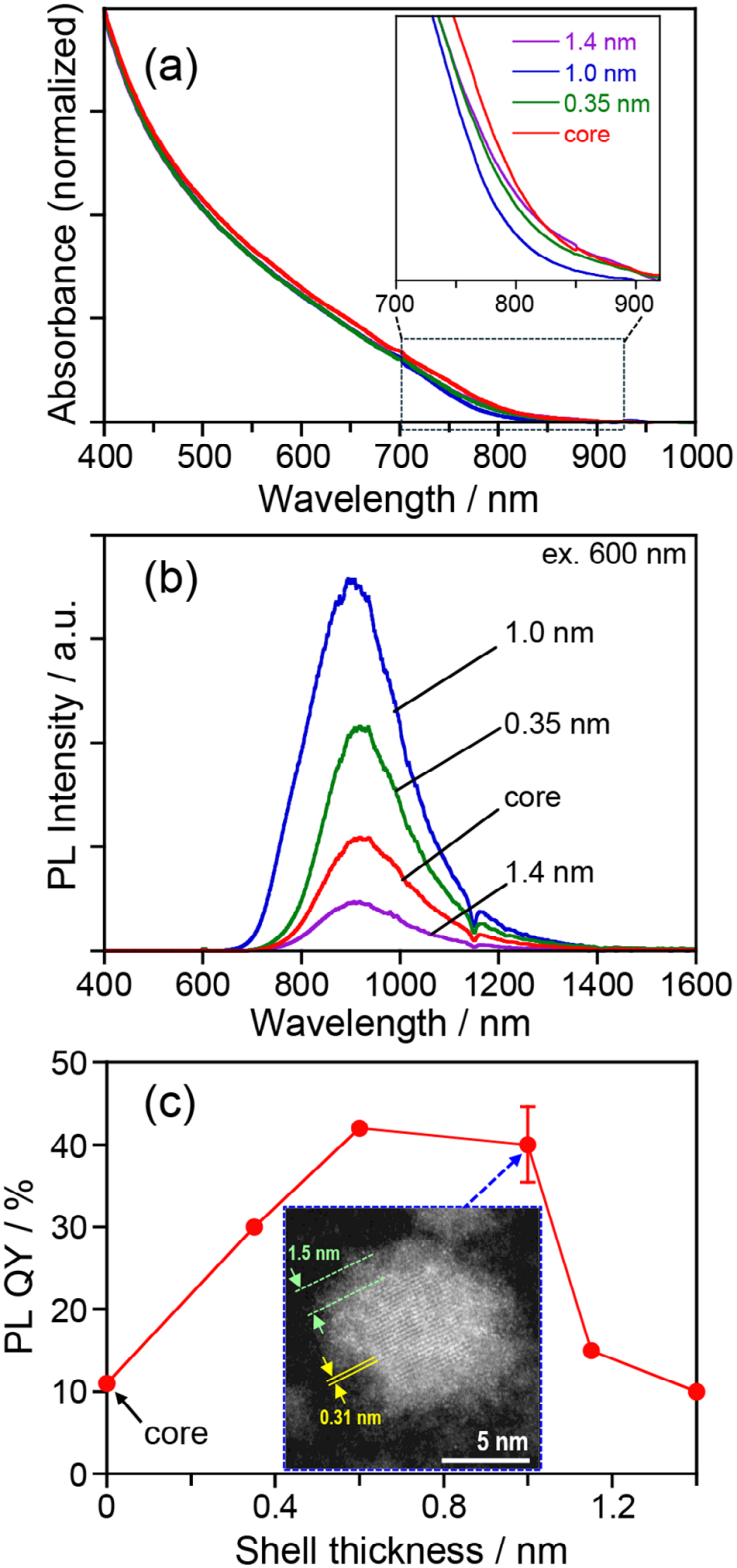
Absorption spectra a), PL spectra b), and PL QY values c) of Ag_8_GeS_6_ core and Ag_8_GeS_6_@ZnS core‐shell QDs with different shell thicknesses. The Ag_8_GeS_6_ QDs used as a core were prepared with Ge/(Ag+Ge) = 0.82. The inset of panel c shows a HAADF‐STEM image of an Ag_8_GeS_6_@ZnS core‐shell QD of 1.0 nm in ZnS shell thickness.

We chose Ag_8_GeS_6_ QDs prepared with Ge/(Ag+Ge) = 0.82 in the precursors as a core to prepare core‐shell QDs because of the highest PL QY (11%). Coating a ZnS layer was carried out with two different preparation strategies. One strategy was a heating‐up method that involved heating an OLA solution containing the core QDs and the ZnS precursors. The other strategy was dropwise addition of a ZnS precursor solution with different injection rates. Figure S7 (Supporting Information) shows TEM images of thus‐obtained Ag_8_GeS_6_@ZnS QDs. Spherical particles were formed regardless of the conditions of ZnS precursor addition. The *d*_av_ value of Ag_8_GeS_6_@ZnS core‐shell QDs obtained by the former method was increased to 5.0 nm from that of core QDs used, 4.3 nm. On the other hand, the latter method more remarkably increased the *d*_av_ of resulting QDs: Decreasing the addition rate of the ZnS precursor solution from 0.075 to 0.016 cm^3^/min increased the *d*_av_ value from 5.5 nm to 7.1 nm, indicating that the formation of pure ZnS nuclei was inhibited by maintaining a lower concentration of ZnS precursors. Consequently, a larger amount of ZnS was deposited on the surfaces of preexisting QDs, resulting in a thicker shell layer. It can be assumed that the shell thickness was equal to half the difference between the *d*_av_ values before and after ZnS shell coating. We found that a very thin ZnS shell was formed on core QDs but that the shell thickness was controllable from 0.35 nm to 1.4 nm, depending on the conditions of ZnS shell formation (Figure S7g, Supporting Information), with a thinner ZnS shell being formed by a higher injection rate of the ZnS precursor solution. XRD measurements of Ag_8_GeS_6_@ZnS QDs (Figure S8, Supporting Information) revealed that the obtained core‐shell QDs exhibited broad diffraction peaks similar to those obtained with core QDs of the argyrodite Ag_8_GeS_6_ crystal structure, but no peaks were assignable to a wurtzite ZnS crystal structure. The main peak observed with Ag_8_GeS_6_@ZnS QDs appeared at almost constant 2*θ* angles, ca. 29°, regardless of the presence of a ZnS shell or its thickness, indicating that the added ZnS did not make alloying with the core material, Ag_8_GeS_6_. The formation of a core‐shell structure of Ag_8_GeS_6_@ZnS QDs with a shell thickness of 1.0 nm was confirmed by using HAADF‐STEM imaging. As shown in **Figures** **5**c inset and Figure S9 (Supporting Information), Ag_8_GeS_6_ cores showed well‐defined lattice fringes with an interplanar spacing of 0.31 nm, corresponding to the (0 2 2) plane of an argyrodite Ag_8_GeS_6_ structure, which has a reported spacing of 0.305 nm. The surfaces of Ag_8_GeS_6_ nanocrystals were coated with amorphous shell layers, thicknesses of which were 1–1.5 nm, being roughly consistent with that observed from the size difference before and after ZnS shell formation. It should be noted that thin shell layers exhibited lower contrast than that of the Ag_8_GeS_6_ cores in HAADF‐STEM images, indicating that the shells contained elements with a lower atomic number, such as Zn, than the heavier Ag and Ge present in the cores. Furthermore, no additional peaks appeared in the XRD patterns even after coating the Ag_8_GeS_6_ QDs with ZnS (Figure S8, Supporting Information). An amorphous thin layer around the Ag_8_GeS_6_ QD core was not observed before ZnS coating as shown in Figure 2a. These results suggest that the shell layer deposited around Ag_8_GeS_6_ QDs consisted of amorphous Zn‐rich layer, such as such as ZnS or Zn‐doped Ag_8_GeS_6_. Furthermore, compositional analysis with XPS, which is highly sensitive to surface composition, confirmed the presence of the Zn‐rich layer on the particle surface, as discussed below.

The XPS spectra shown in Figure S10 (Supporting Information) provide key insights into the chemical environment of the Ag_8_GeS_6_ QDs and the Ag_8_GeS_6_@ZnS core‐shell QDs, as evidenced by the binding energy profiles of emitted photoelectrons. The Ag 3d_5/2_ and 3d_3/2_ peaks of Ag_8_GeS_6_ QDs appeared at 367.14 and 373.24 eV, corresponding to the Ag^+^ sulfidation state characteristic of Ag–S bonds in Ag‐based chalcogenides.^[^ ^52^, ^53^ ^]^ After coating with a ZnS shell, these Ag 3d peaks shifted slightly to lower binding energies at 367.05 and 373.15 eV, suggesting an increase in the electron density of Ag in Ag_8_GeS_6_@ZnS QDs due to electron transfer from Zn to Ag (electronegativity: Ag 1.93, Zn 1.65).^[^ ^54^ ^]^ The Ge 3d peaks of both samples (Ag_8_GeS_6_: 30.14 and 31.64 eV, Ag_8_GeS_6_@ZnS: 30.35 and 31.85 eV) corresponded to Ge^4+^ sulfidation states arising from Ge–S bonds in Ge‐based chalcogenides.^[^ ^55^, ^56^ ^]^ In the S 2p spectra of Ag_8_GeS_6_ QDs, lower binding energy peaks at 160.84 and 162.04 eV were assigned to S 2p_3/2_ and 2p_1/2_, respectively, for the sulfides (S^2−^) bonded to Ag and Ge within the Ag_8_GeS_6_ crystal lattice. Higher binding energy peaks, fitted at 163.04 and 164.24 eV, were attributed to S 2p_3/2_ and 2p_1/2_ of C–S bonds, respectively, originating from DDT bound to the surface of QDs and/or physically adsorbed DDT molecules.^[^ ^57^, ^58^ ^]^ After the core‐shell formation, the higher binding energy peaks disappeared, indicating the removal of surface‐bound DDT molecules due to ZnS coverage. Furthermore, Zn 2p_3/2_ and 2p_1/2_ peaks were observed at 1020.95 and 1043.45 eV, respectively, only after core‐shell formation, corresponding to Zn^2+^ sulfidation states.^[^ ^59^ ^]^ Based on these findings, the valence states of the elements in the QDs were determined as +1 for Ag, +4 for Ge, −2 for S, and +2 for Zn, consistent with the expected valence states in Ag_8_GeS_6_ and Ag_8_GeS_6_@ZnS QDs. The chemical composition of Ag_8_GeS_6_@ZnS QDs, as determined by XPS measurements (Table S2, Supporting Information), revealed a higher Zn content than that indicated by EDS analysis. Since XPS analysis is highly sensitive to the surface composition, this discrepancy confirmed that a Zn‐rich layer such as a ZnS shell was deposited on the surface of Ag_8_GeS_6_ cores.

Figure 5a shows the absorption spectra of Ag_8_GeS_6_@ZnS QDs with different shell thicknesses. The absorption onsets were almost constant at ca. 810 nm regardless of the ZnS shell thickness, suggesting that the size and composition of Ag_8_GeS_6_ cores were not changed by the ZnS shell coating. On the other hand, Ag_8_GeS_6_@ZnS QDs exhibited a broad PL peak (Figure 5b) and had a large Stokes shift of ca. 100 nm between the absorption onset and the PL peak. The wavelength of the PL peak was slightly blue‐shifted from 920 nm to 900 nm with an increase in the ZnS shell thickness from 0 to 1.0 nm but was inversely red‐shifted to 920 nm with a further increase in the shell thickness to 1.4 nm. The PL intensity was increased with an increase in the ZnS shell thickness up to 1.0 nm. It was reported that bulk Ag_8_GeS_6_ exhibited PL at very low temperatures, <130 K, in which a narrow peak assignable to band‐edge emission and a wider PL peak originating from defect sites appeared at 1.57 eV (790 nm) and 1.35 eV (920 nm), respectively, though no PL peak was observed at room temperature.^[^ ^38^ ^]^ Thus, it was suggested that the observed PL peaks of Ag_8_GeS_6_ QDs were attributed to defect‐site emission, regardless of the thickness of the ZnS shell.

Figure 5c shows the relationship between the PL QY of Ag_8_GeS_6_@ZnS QDs and thickness of the ZnS shell. Although Ag_8_GeS_6_ QDs without a shell coating exhibited a moderate PL QY of 11%, the ZnS coating remarkably increased the PL QY: High PL QYs of 42% and 40% were obtained for Ag_8_GeS_6_@ZnS with shell thicknesses of 0.63 nm and 1.0 nm, respectively, but the PL QY decreased with further increase in thickness of the ZnS shell. These results suggested that the deposited ZnS shells effectively removed non‐radiative recombination sites on the surface of the Ag_8_GeS_6_ core and confined photogenerated carriers in the cores by the formation of a type‐I heterostructure, leading to the enlargement of PL intensity. However, an excess amount of ZnS deposition again resulted in the formation of defect sites, causing non‐radiative recombination, probably due to accumulation of the strain between the Ag_8_GeS_6_ core and the ZnS shell.

To compare our results with previously reported low‐toxicity Ag‐based QDs, Table S3 (Supporting Information) summarizes the average size, PL peak wavelength, PL QY, PL lifetime, and typical applications of various NIR‐emitting QDs. Ag_2_S and other Ag‐based multinary semiconductor QDs with low toxicity often suffered from weak emission intensities and lower PL QYs, except for Ag–Au–Se QDs that exhibited an NIR PL peak at 978 nm with PL QY of 65.3%.^[^ ^28^ ^]^ In comparison, the present Ag_8_GeS_6_@ZnS QDs achieved a PL QY of up to ca. 40%, markedly exceeding those of most reported NIR‐emitting QDs, particularly those used for in vivo imaging. This high PL QY can enhance PL brightness in biological media, leading to improved image contrast, higher detection sensitivity, and deeper tissue penetration for bioimaging applications.^[^ ^60^ ^]^

**Figure 6:**
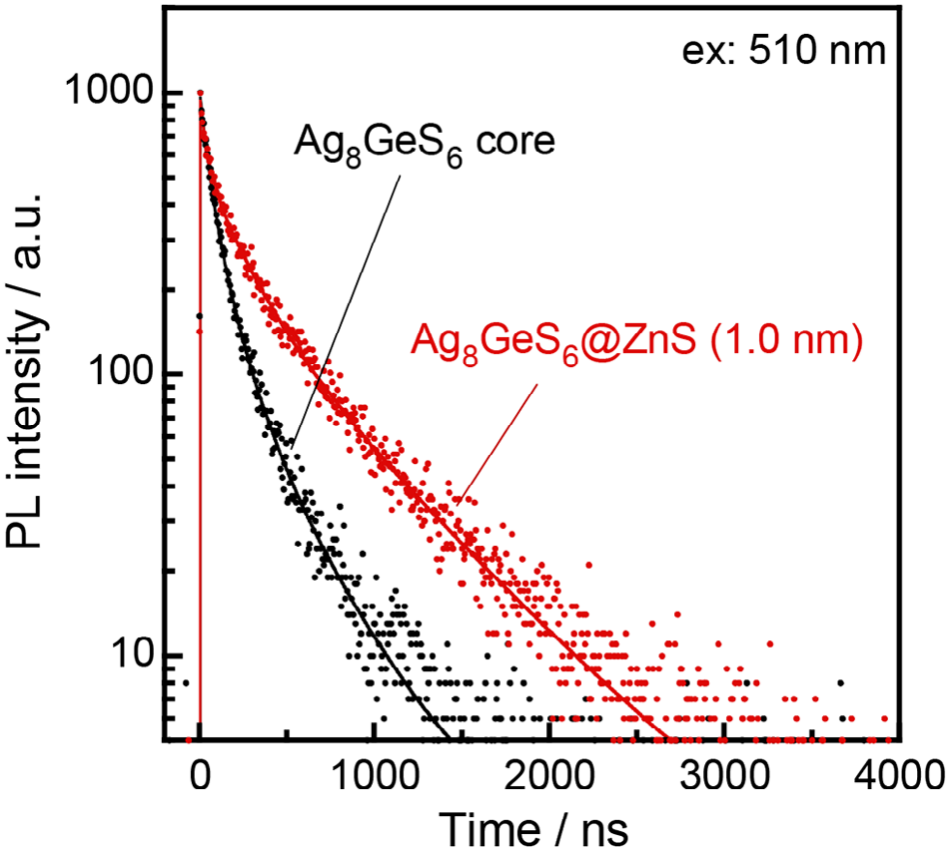
Photoluminescence decay profiles of Ag_8_GeS_6_ (black) and Ag_8_GeS_6_@ZnS with a ZnS shell thickness of 1.0 nm (red). The Ag_8_GeS_6_ QDs used as a core were prepared with Ge/(Ag+Ge) = 0.82. The experimentally obtained curves were fitted by three‐component exponential decay curves (solid lines) with the parameters presented in Table S4 (Supporting Information).

We measured the PL lifetimes to obtain a better understanding of the exciton recombination process. **Figure** **6** shows the decay profiles of Ag_8_GeS_6_ QDs prepared with Ge/(Ag+Ge) = 0.82 and Ag_8_GeS_6_@ZnS QDs with a shell thickness of 1.0 nm, which were recorded at the PL peak wavelength of each sample. The PL decay profiles were fitted with the following three‐component exponential Equation (2):

(2) $$I\left(t\right)=\sum_{n=1}^{3}A_{n}\exp\left(-t/\tau_{n}\right)$$

where τ_*n*_ represents the decay lifetime of PL emission and *A_n_* represents the amplitude corresponding to the lifetime. The fitting results are summarized in Table S4 (Supporting Information). The average lifetime, <τ>, was calculated by using equation (3):

(3) $$\langle \tau \rangle =\frac{\sum_{n=1}^{3}A_{n}\tau_{n}^{2}}{\sum_{n=1}^{3}A_{n}\tau_{n}}$$

The average lifetimes were estimated to be 213 ns for Ag_8_GeS_6_ QDs and 486 ns for Ag_8_GeS_6_@ZnS QDs. The observed PL lifetimes were shorter than those reported for Ag_2_S QDs (Table S3, Supporting Information), which generally exhibited lower PL QYs. It was reported that the lifetimes of band‐edge emission were in a time region of ca.10–100 ns for visible‐light PL of Cd‐based II–VI semiconductor QDs such as CdTe^[^ ^61^, ^62^ ^]^ and CdSe^[^ ^63^, ^64^, ^65^ ^]^ as well as for near‐IR PL of Zn–Ag–In–Te QDs,^[^ ^66^ ^]^ while the lifetimes of defect‐site emission were ca. >500 ns for I–III–IV chalcopyrite semiconductor QDs such as Zn–Ag–In–S^[^ ^67^ ^]^ and Zn–Cu–In–S^[^ ^68^ ^]^ QDs. Thus, the longer PL lifetimes of the present QDs indicated that the PL originated from defect sites regardless of the presence of a ZnS shell. It should be noted that the average lifetime of Ag_8_GeS_6_@ZnS QDs was about twice longer than that of Ag_8_GeS_6_ QDs. These findings suggested that defect states, acting as non‐radiative recombination sites, were present on the surface of Ag_8_GeS_6_ cores and that the coating of the cores with ZnS effectively removed such defect sites, resulting in extension of their carrier lifetime.

The radiative recombination rate constant (*k_rad_*) and non‐radiative recombination rate constant (*k_nr_*) can be estimated from the average lifetime and PL QY. The total recombination rate is given by:

(4) $${\langle \tau \rangle }^{-1}=k_{rad}+k_{nr}$$

Additionally, the PL QY is related to these rate constants by the following equation^[^ ^28^, ^69^ ^]^:

(5) $$PL\;QY=\frac{k_{rad}}{k_{rad}+k_{nr}}$$

We obtained *k*_nr_ values of 4.2 × 10^6^ s^−1^ and 1.2 × 10^6^ s^−1^ and *k*_rad_ values of 0.52 × 10^6^ s^−1^ and 0.82 × 10^6^ s^−1^ for Ag_8_GeS_6_ QDs and Ag_8_GeS_6_@ZnS QDs, respectively. Although the *k*_nr_ value of Ag_8_GeS_6_ QDs decreased to about one‐fourth due to the surface coating with a ZnS shell, the *k*_rad_ value increased by 1.6 times as a result of the ZnS coating. These results indicated that non‐radiative defect sites on the Ag_8_GeS_6_ surface were eliminated by the surface coating with a ZnS shell, resulting in the enhancement of PL originating from radiative defect sites inside the crystal.

### 3.3 Application of Ag_8_GeS_6_@ZnS QDs to In Vivo NIR Imaging

The obtained Ag_8_GeS_6_@ZnS QDs emitted PL with a relatively high QY in the ranges of the first biological window of 700–950 nm (NIR‐I) and the second biological window of 1000–1350 (NIR‐II) as mentioned above, making them suitable for a bioimaging probe. One key challenge for this achievement is the improvement of the dispersibility of QDs in aqueous solutions without loss of their PL property, because the obtained QDs were hydrophobic due to the surface modification with OLA and DDT as capping ligands. To evaluate the potential ability of Ag_8_GeS_6_@ZnS QDs as an NIR luminescent probe for in vivo bioimaging, we carried out a ligand exchange process using hydrophilic MPA molecules. The introduction of MPA can confer a hydrophilic nature on the surface of QDs, enabling uniform dispersion of resulting QDs in aqueous solutions and improving their biocompatibility. It was reported that the dynamics of ligand exchange on the QD surface could be described as an adsorption‐desorption equilibrium process,^[^ ^70^ ^]^ in which MPA had a high affinity to the surface and replaced original capping ligands such as OLA and DDT on QDs.

**Figure 7:**
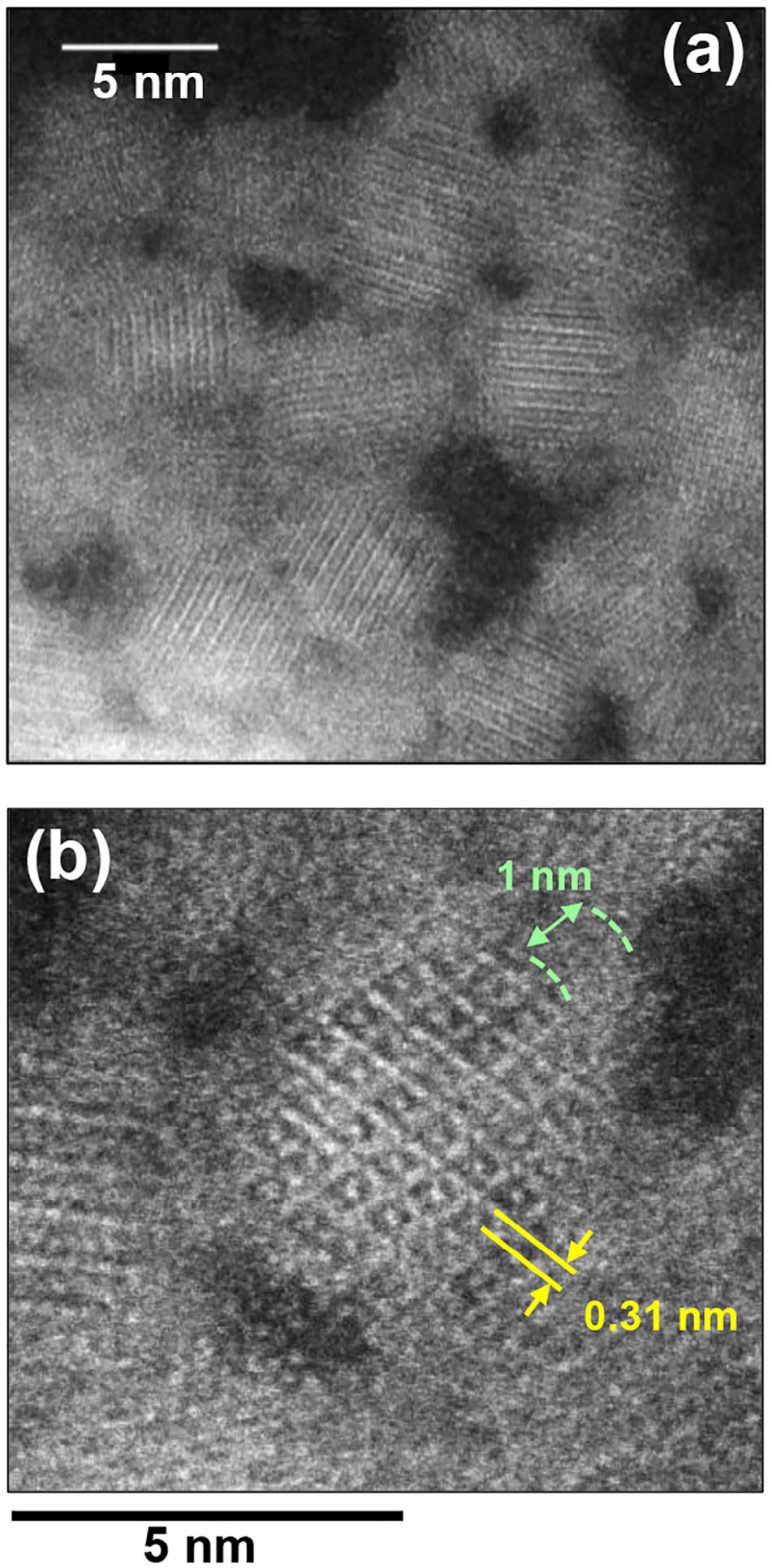
Representative HAADF‐STEM images of Ag_8_GeS_6_@ZnS‐MPA QDs with a ZnS shell thickness of 1.0 nm. Images are a) a wide area and b) a high‐magnification image.

The thus‐obtained Ag_8_GeS_6_@ZnS‐MPA QDs were uniformly dispersed in an aqueous solution. Figure S11 (Supporting Information) shows TEM images of Ag_8_GeS_6_@ZnS‐MPA QDs. The average size and distribution of QDs remained almost unchanged after ligand exchange: The *d*_av_ values remained constant at 6.3 nm, regardless of the type of surface ligand, and the standard deviations (σ) were 0.54 and 0.59 nm before and after MPA modification, respectively. On the other hand, the average hydrodynamic size of Ag_8_GeS_6_@ZnS‐MPA QDs dispersed in water, determined by dynamic light scattering, was 13.9 nm (Figure S12, Supporting Information), being slightly larger than that determined by TEM measurements. This increase in hydrodynamic size was probably due to the presence of hydrated MPA ligands and/or a solvation layer surrounding the QDs in water, which were not visible in TEM measurements conducted under dry conditions. The observed hydrodynamic size is sufficiently small and appears suitable for in vivo bioimaging applications, as it falls within the optimal size range for enhanced tissue penetration and efficient renal clearance, thereby minimizing the risk of organ accumulation.^[^ ^71^, ^72^ ^]^ **Figure** **7** shows high‐resolution HAADF‐STEM images of Ag_8_GeS_6_@ZnS‐MPA with a ZnS shell thickness of 1.0 nm. The obtained QDs were spherical and well‐dispersed, indicating that the ligand exchange process hardly changes the nanostructure of original QDs. Each QD had clear lattice fringes in the core region without showing a grain boundary, in which the interplanar spacing was determined to be 0.31 nm attributed to the (0 2 2) plane of an orthorhombic Ag_8_GeS_6_ crystal. These findings indicated that the cores were composed of highly crystalline Ag_8_GeS_6_. Furthermore, the surface of Ag_8_GeS_6_ cores was covered with an amorphous layer with a thickness of ca. 1 nm, which was in good agreement with the shell thickness estimated from the difference between the average diameters before and after ZnS coating. These results indicated that an amorphous Zn‐rich layer, such as ZnS or Zn‐doped Ag_8_GeS_6_, on the Ag_8_GeS_6_ core, was not significantly changed by the ligand exchange. During the HAADF‐STEM measurement, we conducted nanoscale EDS analysis for Ag_8_GeS_6_@ZnS‐MPA QDs. The results of the nanoscale EDS analysis revealed distinct chemical compositions in the center and surface regions of Ag_8_GeS_6_@ZnS‐MPA QDs. The chemical composition near the QD surface was Ag/Ge/S/Zn = 63.0/3.6/31.6/1.9, while the composition close to the central area of the QD was 65.6/4.9/29.4/0.1. The fractions of Zn and S on the surface of the QDs were found to be larger than those in the core of the QDs, indicating that the surface layer contained a remarkably larger fraction of Zn due to the presence of a Zn‐rich layer on the Ag_8_GeS_6_ core. This observation strongly suggests that the core/shell structure of the QDs remained intact even after the ligand exchange process. It should be noted that the ratio of Ge/(Ag+Ge)_QD_ detected in the central area of individual QDs during the nanoscale EDS measurements was 0.070, being significantly smaller than that obtained from ensemble EDS analysis of Ag_8_GeS_6_@ZnS‐MPA QDs, Ge/(Ag+Ge)_QD_ = 0.17 (Table S2, Supporting Information). This discrepancy was likely caused by damage to the QDs from the high‐energy electron beam used in STEM measurements, which can induce the sputtering of constituent atoms from the QDs.

**Figure 8:**
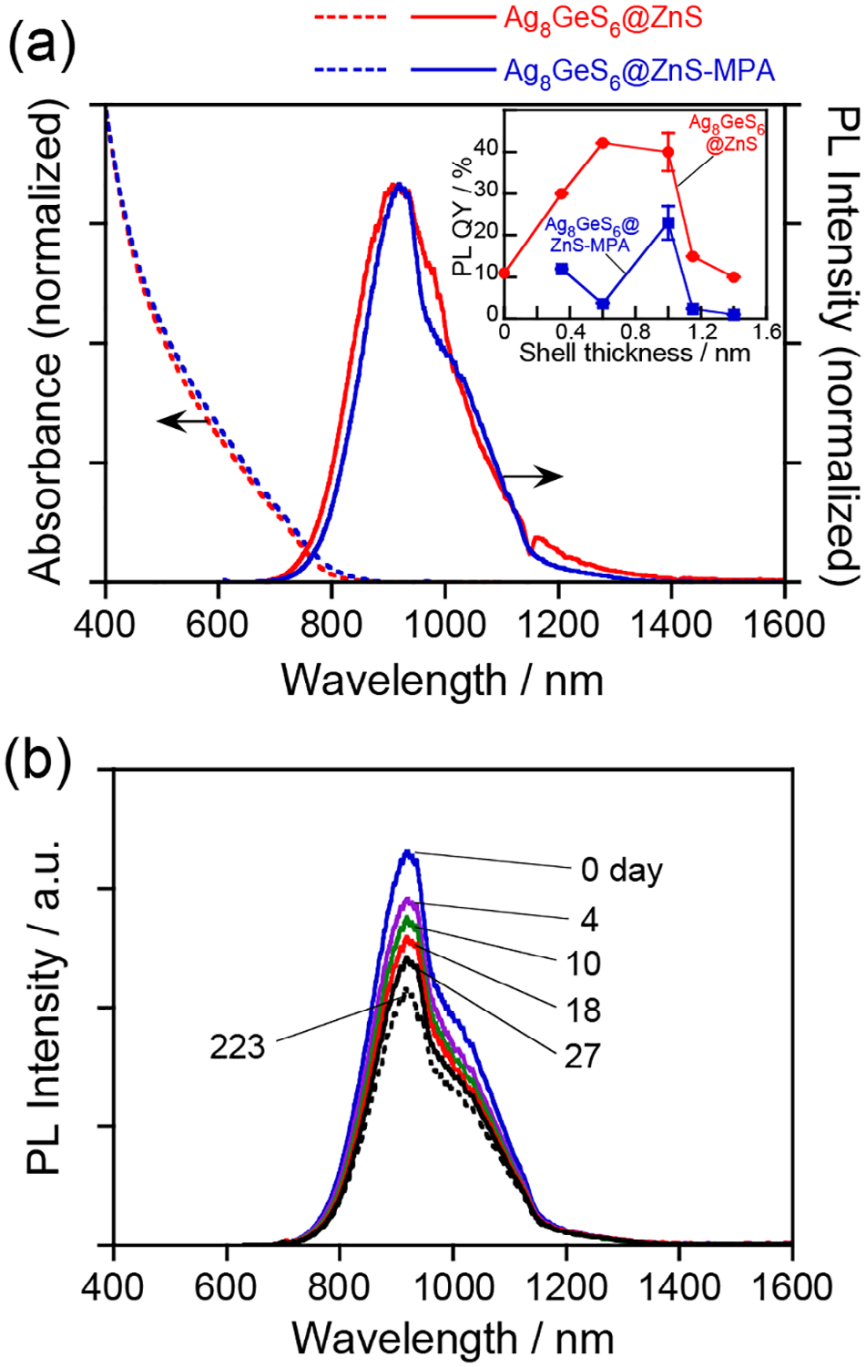
a) Absorption and PL spectra of Ag_8_GeS_6_@ZnS and Ag_8_GeS_6_@ZnS‐MPA QDs. The ZnS shell thickness of QDs was 1.0 nm. (Inset) Changes in PL QYs of Ag_8_GeS_6_@ZnS QDs before (red) and after ligand exchange with MPA (blue) as a function of the ZnS shell thickness. b) Changes in PL spectra of Ag_8_GeS_6_@ZnS‐MPA QDs uniformly dispersed in aqueous solutions. The solution was stored in the dark at 4 °C under an N_2_ atmosphere. The numbers in the panel are the storage time in the unit of day. The wavelength of excitation light was 600 nm.

**Figure** **8**a shows the changes in optical properties of Ag_8_GeS_6_@ZnS with a shell thickness of 1.0 nm, observed before and after ligand exchange with MPA. The absorption spectra remained unchanged, with the onset wavelength being 810 nm. The profiles of PL spectra were almost consistent, except that the peak wavelength slightly shifted from 900 to 920 nm after MPA modification and a dip at ≈1000 nm appeared for the PL spectrum of Ag_8_GeS_6_@ZnS‐MPA due to light absorption with water. However, the PL QY decreased from 40% to 23% after the MPA modification. Since the fractions of Ag and Ge became smaller for Ag_8_GeS_6_@ZnS‐MPA QDs (Table S2, Supporting Information), probably due to leaching during the ligand exchange process, the change in the chemical composition and/or the adsorption of water molecules on the QD surface produced non‐radiative recombination pathways, resulting in deterioration of the PL QY of the QDs. As shown in the inset of Figure 8a, the degree of PL QY decrease with MPA modification was also dependent on the ZnS shell thickness. Although the PL QYs with QDs having ZnS shell thicknesses of 0.63 and 1.0 nm were similar at ≈40–42% before ligand exchange, the MPA modification decreased the PL QYs to 4% and 23% for Ag_8_GeS_6_@ZnS‐MPA QDs with shell thicknesses of 0.63 nm and 1.0 nm, respectively. This suggested that a thicker ZnS shell better protected the QD cores from damage during the ligand exchange. It is notable that despite the decrease in the PL QY, the MPA‐modified QDs with a ZnS shell thickness of 1.0 nm still exhibited a relatively high QY, 23%, in water, compared to those previously reported for low‐toxicity multinary QDs, such as Cu–In–S,^[^ ^73^ ^]^ Zn–Ag–In–S,^[^ ^74^ ^]^ Zn–Cu–In–S,^[^ ^75^ ^]^ Ag–In–Ga–Se and Ag–In–Ga–S–Se.^[^ ^23^, ^24^, ^29^ ^]^ Thus, we selected Ag_8_GeS_6_@ZnS‐MPA with a shell thickness of 1.0 nm for evaluating the potential applicability of these QDs for bioimaging.

**Figure 9:**
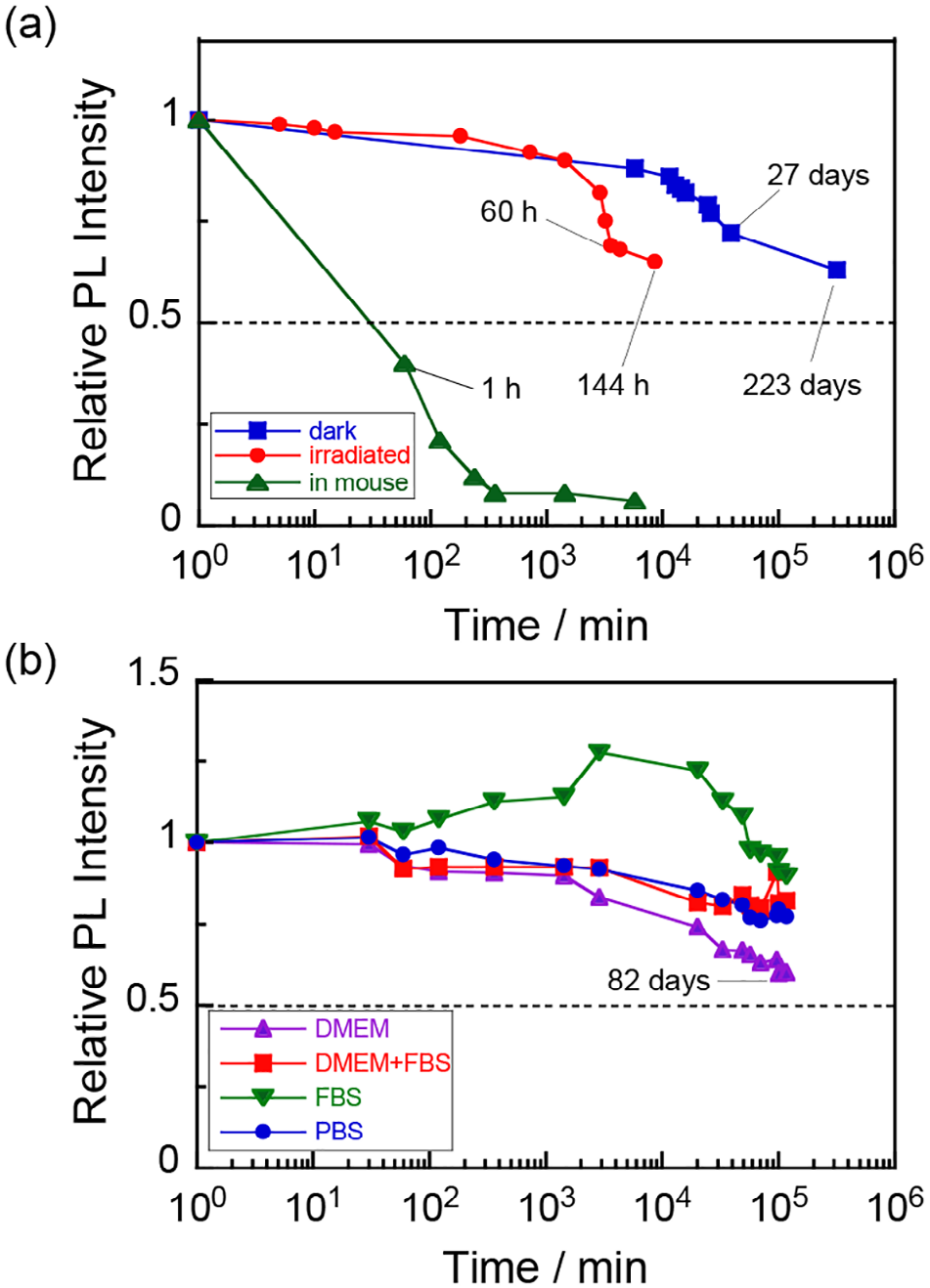
Time courses of PL intensity of Ag_8_GeS_6_@ZnS‐MPA in various solutions. a) The QDs in pure water were stored in the dark at 4 °C (blue) and by irradiation with monochromatic light at 700 nm (intensity: 4.5 mW cm^−2^) (red). b) The QDs were dispersed in various types of biological media in the dark at 4 °C. In panel a, the change in PL intensity of Ag_8_GeS_6_@ZnS‐MPA injected into the back of a mouse (green) is also shown. The measurements started at the 1st min. Each PL intensity was normalized by the corresponding initial value.

Since the PL stability of the QDs is an important parameter for bioimaging application, we evaluated the PL stability of Ag_8_GeS_6_@ZnS‐MPA QDs in water under various conditions. Figure 8b shows the PL spectra of Ag_8_GeS_6_@ZnS‐MPA QDs in an N_2_ atmosphere. The PL intensity monotonously decreased with the elapse of storage time, while the peak shape was almost unchanged and no additional PL peaks emerged. This observation indicated that defect sites, acting as non‐radiative recombination sites but not as radiative ones, were formed on the surface of the Zn‐rich layer (ZnS or Zn‐doped Ag_8_GeS_6_) or in the Ag_8_GeS_6_ core during storage. **Figure** **9**a shows the changes in relative PL peak intensity at 920 nm of the QDs with elapse of storage time. Storing the QD aqueous solution in the dark at 4 °C under an N_2_ atmosphere gradually reduced the PL intensity: The PL intensity retained more than 70% of its initial value even after 27 days of storage, and the QDs exhibited 60% of the initial PL intensity of the solution even after 223 days. In contrast, the PL intensity was more rapidly deteriorated when the QD solution was irradiated with monochromatic light at 700 nm of 4.5 mW cm^−2^ in intensity at room temperature under an N_2_ atmosphere (Figure S13, Supporting Information): Within the first 60 h, the PL intensity decreased to 70% of its initial value. TEM measurements revealed that the Ag_8_GeS_6_@ZnS‐MPA QDs were aggregated after the PL stability tests, as shown in Figure S14 (Supporting Information). This suggested that a slow desorption of MPA ligands from the QD surface occurred in the dark to aggregate the QDs in an aqueous solution, resulting in exposure of the bare QD surface, which induced the formation of non‐radiative recombination sites. Such desorption of MPA was also accelerated by the irradiation, because photogenerated holes in QDs oxidized MPA ligands adsorbed on the surface to expose the bare surface of QDs. It has been reported for low‐toxicity multinary QDs that the half‐life periods of PL intensity in the dark under an N_2_ atmosphere were 15.5 days and 15 days for Ag–In–Ga–Se@GaS_x_^[^ ^23^ ^]^ and Ag–In–Ga–S–Se@GaS_x_^[^ ^29^ ^]^ QDs incorporated in liposomes, respectively. In contrast, Ag_8_GeS_6_@ZnS‐MPA QDs in water had a much longer half‐life period, >223 days, showing that the ZnS shell was more stable than the GaS_x_ shell in water.

The PL stability of Ag_8_GeS_6_@ZnS‐MPA QDs in various biological media was further evaluated over an 82‐day period to assess the impact of the surrounding environment on their PL properties. The initial PL intensities of QDs in biological media were significantly reduced compared to those in pure water, as shown in Figure S15 (Supporting Information). The QDs diluted with DMEM, DMEM with 10% FBS (DMEM‐FBS), and PBS exhibited about half the PL intensity of that in water, while the QDs diluted with FBS showed only one‐seventh of the PL intensity in water. This reduction in PL intensity was probably due to the adsorption of ionic and/or organic species, such as phosphate ion, amino acids, vitamins, and proteins, on the QD surface, causing changes in size or surface modifications.^[^ ^76^ ^]^　During the durability tests, the PL peak wavelength remained unchanged. However, as shown in Figure 9b, the PL intensities of the QDs decreased with the elapse of storage, the degree depending on the type of the media. After 82 days of storage, the QDs retained 60% of the initial PL intensity in DMEM, 82% in DMEM‐FBS, and 77% in PBS. In contrast, the QDs in FBS showed the highest retention, maintaining 90% of their initial PL intensity, though the initial PL intensity in FBS was significantly lower than those in the other media. Although the retention of PL intensity varied across different media, the QDs exhibited substantial PL stability over a period of 82 days.

**Figure 10:**
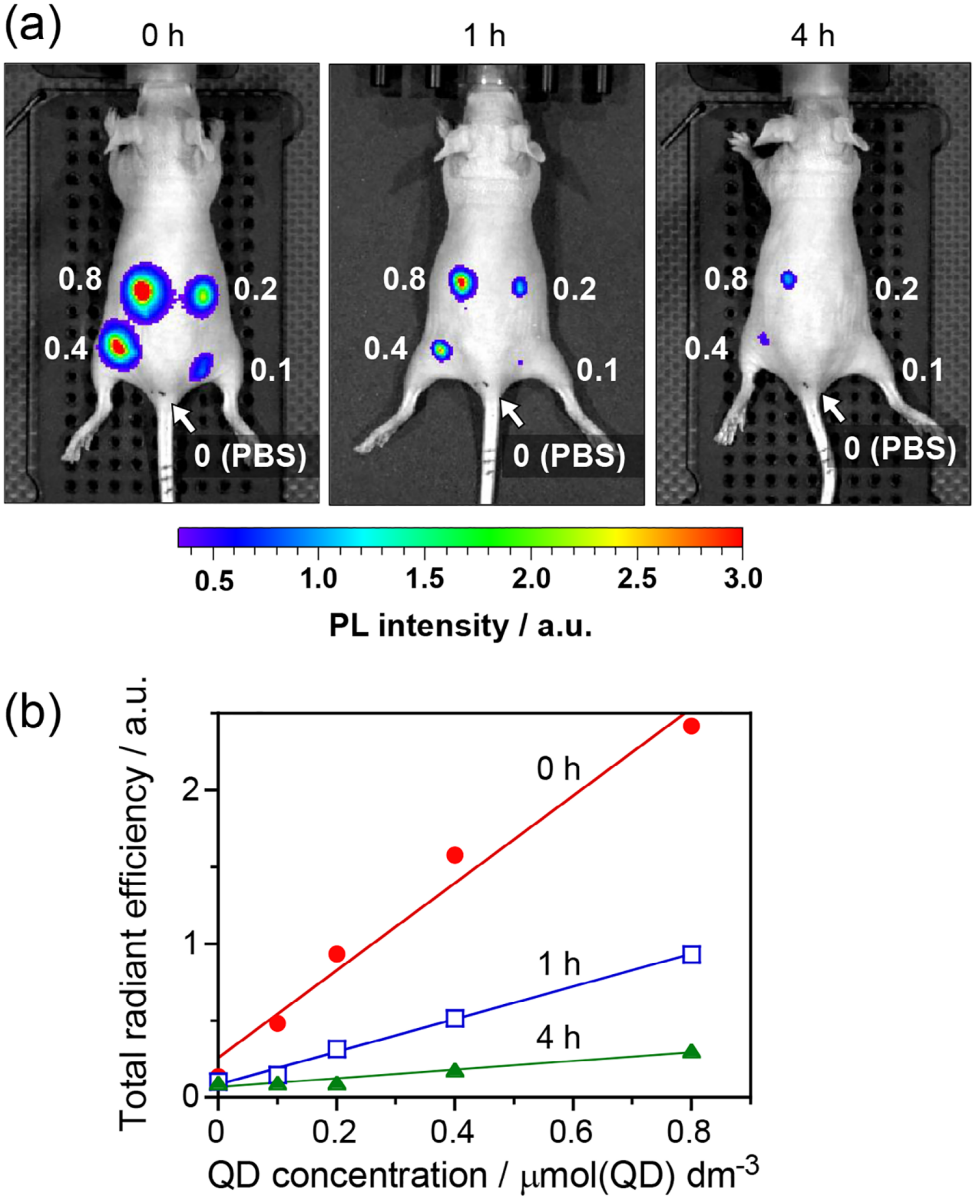
In vivo PL images of a mouse subcutaneously injected with Ag_8_GeS_6_@ZnS‐MPA dispersions (each 50 mm^3^) in the back at 0 h, 1 h, and 4 h a). The numbers in the panels are the concentrations of QDs in the dispersions in the unit of µmol(QDs) dm^−3^. b) Relationships between PL intensities detected through the skin of the mouse at 0 h (red), 1 h (blue), and 4 h (green) and concentrations of Ag_8_GeS_6_@ZnS‐MPA QDs in the solutions injected.

Ag_8_GeS_6_@ZnS‐MPA QDs were subcutaneously injected into the back of a nude mouse after being dispersed in phosphate‐buffered saline (PBS, pH 7.4). **Figures** **10**a and S16 (Supporting Information) show NIR PL images of the QD‐injected mouse as a function of elapsed time. Intense PL emission from the QDs was detected through the skin immediately after the QD injection, and a linear relation was observed between the PL intensity detected and the concentration of QDs injected (Figure 10b). The PL signal from the mouse significantly decreased with the elapse of the time, but PL of Ag_8_GeS_6_@ZnS‐MPA was still detected from the mouse at 24 h and 4 days after injection. Although the detection sensitivity was remarkably reduced, linear relations between the PL intensity and the QD concentration were also observed at 1 h and 4 h after injection.

The PL intensity of Ag_8_GeS_6_@ZnS‐MPA (0.80 µmol(QDs) dm^−3^) injected into the mouse is also plotted in Figure 9a as a function of elapsed time. The rate of decrease in PL intensity was significantly higher in the mouse than in water or biological media. We estimated the half‐life period of PL intensity in the mouse to be ca. 0.5 h by interpolation, being much shorter than the values expected in the solutions under an N_2_ atmosphere, >80 days. These findings indicated that the environmental condition inside living tissue was much more severe for Ag_8_GeS_6_@ZnS‐MPA QDs.

**Figure 11:**
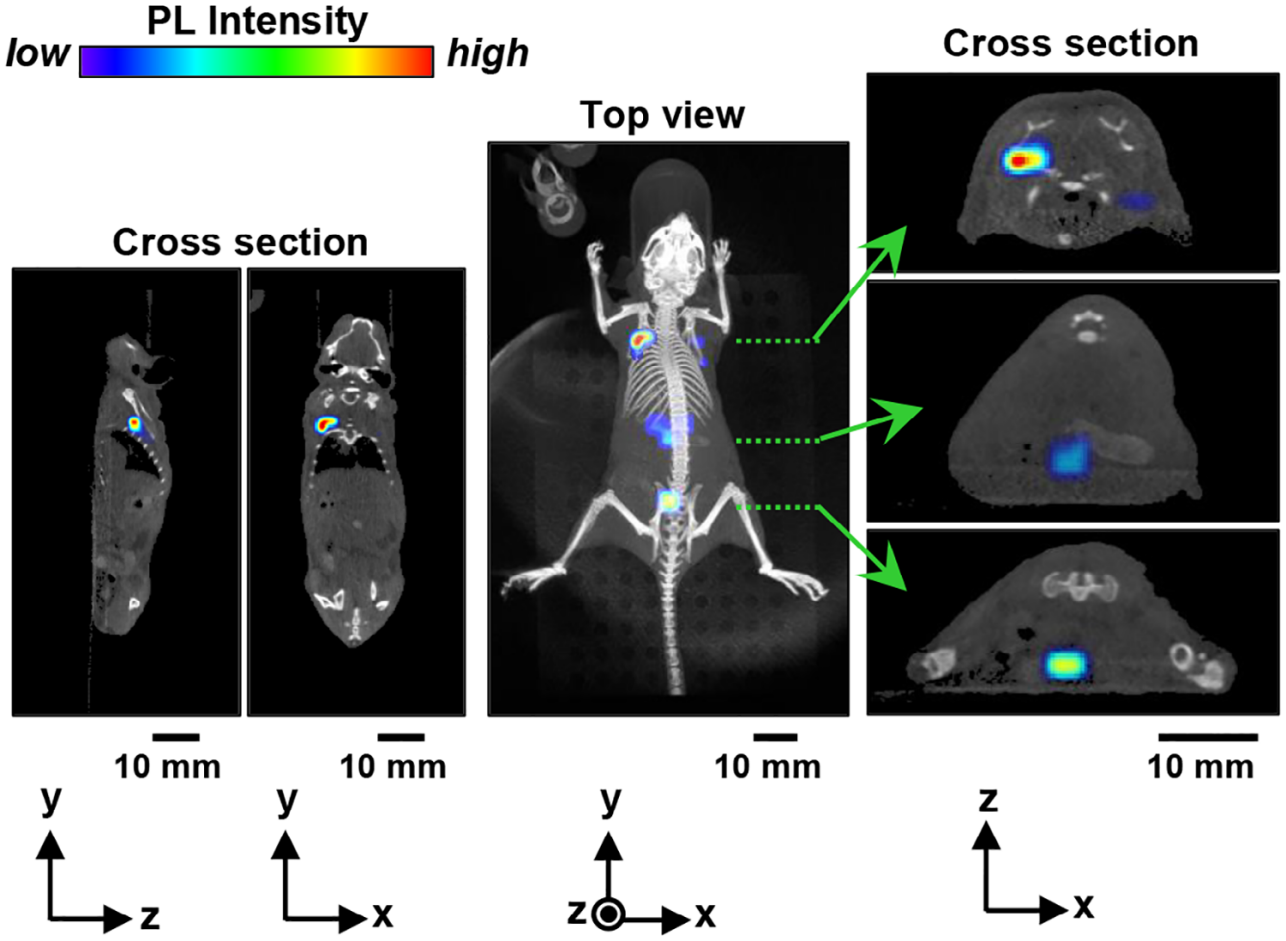
A 3D PL image overlaid onto the corresponding X‐ray CT image of a mouse, taken 4 h after intravenous injection of Ag_8_GeS_6_@ZnS‐MPA QDs into its tail. Distinct PL signals were detected from the lungs (right top), intestines (right middle), and bladder (right bottom).

To evaluate systemic circulation and deep‐tissue imaging, Ag_8_GeS_6_@ZnS‐MPA QDs (0.8 µmol(QDs) dm^−3^) were injected intravenously into a mouse, and their biodistribution was monitored over time (Figure S17, Supporting Information). PL imaging of the mouse revealed that the QDs were carried by the bloodstream, circulating throughout the entire body within 15 min post‐injection (Movie S1, Supporting Information). At 4 h post‐injection, the injected QDs were accumulated in the lungs, intestines and bladder, indicating active excretion of QDs through hepatic and renal pathways. Since the PL signals of the injected QDs disappeared from the mouse at 24 h post‐injection, it was concluded that the QDs were excreted. The penetration depth of the QD PL signal was assessed in vivo using cross‐sectional imaging, performed 4 h after the QD administration. The organs were located at depths from the back skin of 5.5 mm for the lungs, 11 mm for the intestines, and 15 mm for the bladder, in which PL signals with relatively high intensity were successfully detected (**Figure** **11**; Movie S2, Supporting Information). These results demonstrated that the present imaging system successfully acquired PL signals from tissues at depths of at least 15 mm, highlighting its capability for deep‐tissue imaging.

The in vitro cytotoxicity of Ag_8_GeS_6_@ZnS‐MPA QDs was evaluated using the Cell Counting Kit‐8 (CCK‐8) assay in HeLa cells and Swiss 3T3 cells at various concentrations. After a 24‐h incubation with Ag_8_GeS_6_@ZnS‐MPA QDs, cytotoxicity assessment indicated nearly 100% cell viability in both types of cells for all tested concentrations (up to 30 nmol(QDs) dm^−3^), with no significant differences observed (Figure S18, Supporting Information). These results indicated that Ag_8_GeS_6_@ZnS‐MPA QDs exhibited sufficiently low cytotoxicity for application as an NIR‐luminescent bioimaging probe. This low cytotoxicity was likely due to the presence of the ZnS shell, which effectively prevented the leaching of Ag and Ge in aqueous solutions.

The in vivo biodistribution and toxicity of Ag_8_GeS_6_@ZnS‐MPA QDs were investigated by subcutaneous administration in mice over a period of 14 days. PL imaging showed no significant accumulation of QDs in major organs, including the liver, spleen, kidneys, lungs, heart, and intestines (Figure S19, Supporting Information). Although photoluminescence was observed in the QD‐administered group, the luminescence intensities in these organs were comparable to those in the negative control group injected with PBS, suggesting rapid clearance or minimal retention in any specific tissue (Figure S20, Supporting Information). It was reported that larger QDs tended to accumulate in the liver and spleen due to interactions with the reticuloendothelial system (RES), a network of immune cells responsible for clearing foreign particles from the bloodstream.^[^ ^77^ ^]^ However, the lack of significant accumulation in this study suggested that the small size of Ag_8_GeS_6_@ZnS‐MPA QDs facilitated efficient clearance from the body, possibly through kidney filtration. Additionally, no significant abnormalities were detected in biochemical tests at one week or two weeks after the QD injection. All biochemical values in the QD‐administered group were comparable to those in the control group, with no significant differences observed in the levels of albumin (ALB), aspartate aminotransferase (AST), alanine aminotransferase (ALT), lactate dehydrogenase (LDH), γ‐glutamyl transferase (γ‐GT), and total bilirubin (T‐BIL) – markers commonly associated with liver irritation and potential liver stress due to QD accumulation (Table S5, Supporting Information). These results indicated that no liver stress or damage was present in the treated mice, further supporting the lack of significant accumulation of the QDs in the liver as mentioned previously.

## 4 Conclusion

As an NIR semiconductor, Ag_8_GeS_6_, consisting of only elements with low toxicity, has been investigated for the fabrication of solar energy conversion systems.^[^ ^33^, ^35^, ^36^ ^]^ However, in this study, we found a new photoluminescent property of this material. Through solution‐phase synthesis, we successfully developed a novel type of Ag_8_GeS_6_ QDs exhibiting NIR photoluminescence for the first time. An increase in the Ge/(Ag+Ge) ratio in the precursors slightly increased the Ge fraction of the resulting Ag_8_GeS_6_ QDs without a significant change in the crystal structure of the QDs. The obtained QDs, with an average diameter of 4.2–4.6 nm, had an argyrodite Ag_8_GeS_6_ crystal structure and their energy gaps were almost constant at 1.45 eV regardless of the Ge/(Ag+Ge) ratio in the precursors. In contrast, the PL property of QDs considerably varied with change in the metal precursor ratio: A broad PL peak assignable to defect‐site emission was observed at 920 nm for Ge‐rich Ag_8_GeS_6_ QDs at room temperature, the intensity being enhanced with an increase in the experimentally obtained Ge/(Ag+Ge) ratio in the QDs from 0.11 to 0.21, though Ag_8_GeS_6_ QDs having the stoichiometric ratio of Ge/(Ag+Ge)_QD_, 0.11, showed no detectable photoluminescence. The highest PL QY of 11% was obtained with DDT‐modified Ag_8_GeS_6_ QDs.

The PL property of Ag_8_GeS_6_ QDs was enhanced by surface‐coating with a ZnS shell, and the resulting Ag_8_GeS_6_ core‐ZnS shell QDs had a type‐I heterostructure. The wavelength of the observed PL peak was almost constant at 920 nm, regardless of the presence of the ZnS shell, whereas the PL intensity was remarkably increased with an increase in the thickness of an amorphous ZnS shell up to 1.0 nm. Ag_8_GeS_6_@ZnS QDs with a ZnS shell thickness of 1.0 nm showed a remarkably high PL QY of ≈40%, being one of the best values for previously reported photoluminescent Ag‐based QDs in a similar NIR wavelength range. Since the PL lifetime of the Ag_8_GeS_6_@ZnS QDs was 486 ns, being much longer than the lifetimes reported for band‐edge emission of QDs,^[^ ^78^, ^79^ ^]^ the observed PL peak was assignable to defect‐site emission. Recently, Ag_2_S QDs have also been intensively investigated as an NIR photoluminescent material with low toxicity. It was reported that Ag_2_S QDs with particle diameters of 4–5 nm had *E*_g_ values of ca. 1–1.25 eV, being smaller than those of obtained Ag_8_GeS_6_ QDs, and that they exhibited a broad PL peak at ≈1200 nm with low PL QYs, typically <ca. 2%,^[^ ^80^ ^]^ though the PL QYs of Ag_2_S QDs were increased to ca. 10% by surface treatment with HCl^[^ ^81^ ^]^ or to 15.5% by encapsulation with poly(ethylene glycol) polymer layers.^[^ ^82^ ^]^ Thus, the advantage of ternary Ag_8_GeS_6_ QDs compared to binary Ag_2_S QDs was the remarkable improvement of PL properties by precisely tuning the composition ratio of Ag/Ge of the QDs.

Ligand exchange of hydrophobic surface modifiers of DDT and OLA with MPA enabled uniform dispersion of Ag_8_GeS_6_@ZnS QDs in an aqueous solution, with the PL QY being decreased by this process but maintaining a relatively high value, 23%. The obtained QDs exhibited high PL stability in pure water in the dark under an N_2_ atmosphere, the half‐life period of the PL intensity being longer than 223 days. The obtained Ag_8_GeS_6_@ZnS‐MPA QDs were successfully applied for in vivo bioimaging via both subcutaneous injection and intravenous injection into mice. Clear PL images were obtained through the mouse skin, with the PL intensity being proportional to the concentration of injected QDs. PL signals were acquired from depths of at least 15 mm beneath the back skin, demonstrating the deep‐tissue imaging capability of Ag_8_GeS_6_@ZnS‐MPA QDs. Cytotoxicity assessment revealed high cell viability and safety, indicating their potential for bioimaging applications. The findings obtained in the present study provide valuable insights into the design of less toxic, high‐quality multinary QDs having NIR photoluminescence for a wide range of applications including light energy conversion systems, bioimaging probes, QD‐LEDs and NIR photosensors.

## Conflict of Interest

The authors declare no conflict of interest.

## Author Contributions

N.R., J.K. and T.T. conceived the idea to improve the luminescent properties of QDs by tuning their chemical composition and designed the project. N.R., J.K., M.S., K.A., T.K., T.Y. and T.T. prepared and characterized the QDs. H.Y. and Y.B. applied the QDs to in vivo imaging, evaluated their performance, and conducted a cytotoxicity assessment. N.R., H.Y. and T.T. wrote the paper. All authors discussed the results and commented on the manuscript.

## Supporting information



Supporting Information

Supplemental Movie 1

Supplemental Movie 2

## Data Availability

The data that support the findings of this study are available in the supplementary material of this article.
