# Supplementary material for: Enhancing Near‐Infrared Photoluminescence of Ag8GeS6 Quantum Dots Through Compositional Fine‐Tuning and ZnS Coating for In Vivo Bioimaging
Source: Small. 2025 May 7;21(32):2411142. doi: 10.1002/smll.202411142 (PMC12366261; doi:10.1002/smll.202411142)
Supplement: Supplementary file 1 — Supporting Information [file SMLL-21-2411142-s001.pdf]

NANO · MICRO
small

Supporting Information

for *Small*, DOI 10.1002/smll.202411142

Enhancing Near-Infrared Photoluminescence of Ag_8GeS_6 Quantum Dots Through Compositional Fine-Tuning and ZnS Coating for In Vivo Bioimaging

Nurmanita Rismaningsih, Junya Kubo, Masayuki Soto, Kazutaka Akiyoshi, Tatsuya Kameyama, Takahisa Yamamoto, Hiroshi Yukawa, Yoshinobu Baba and Tsukasa Torimoto**

Supporting Information

Enhancing Near-Infrared Photoluminescence of Ag_8GeS_6 Quantum Dots through Compositional Fine-tuning and ZnS Coating for *In Vivo* Bioimaging

Nurmanita Rismaningsih[1], *Junya Kubo*[1], *Masayuki Soto*[1], *Kazutaka Akiyoshi*[1], *Tatsuya Kameyama*[1], *Takahisa Yamamoto*[1], *Hiroshi Yukawa**[1,2,3,4], *Yoshinobu Baba*[1,2,3], *and Tsukasa Torimoto**[1,2]

[1]Graduate School of Engineering, Nagoya University, Chikusa-ku, Nagoya 464-8603, Japan.
[2]Research Institute for Quantum and Chemical Innovation, Institutes of Innovation for Future Society, Nagoya University, Chikusa-ku, Nagoya 464-8603, Japan.
[3]Institute for Quantum Life Science, National Institutes for Quantum Science and Technology, Anagawa 4-9-1, Inage-ku, Chiba 263-8555, Japan.
[4]Department of Quantum Life Science, Graduate School of Science, Chiba University, Chiba 265-8522, Japan.

E-mails:
torimoto@chembio.nagoya-u.ac.jp (T. Torimoto), yukawa.hiroshi@qst.go.jp (H. Yukawa)

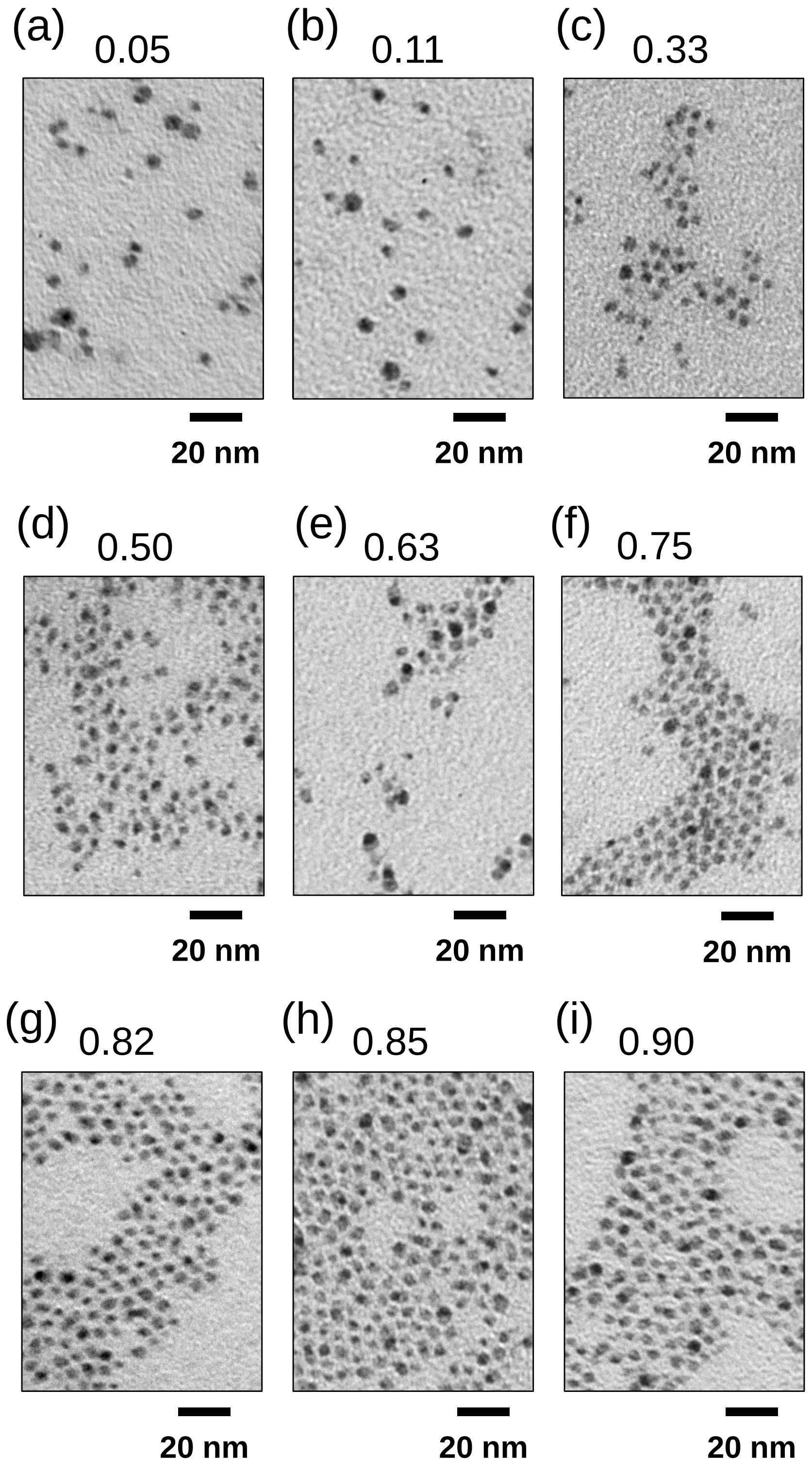

Figure S1. Wide-area TEM images of Ag_8GeS_6 QDs prepared with Ge/(Ag+Ge) = 0.05 (a), 0.11 (b), 0.33 (c), 0.50 (d), 0.63 (e), 0.75 (f), 0.82 (g), 0.85 (h), and 0.90 (i) in the precursors.

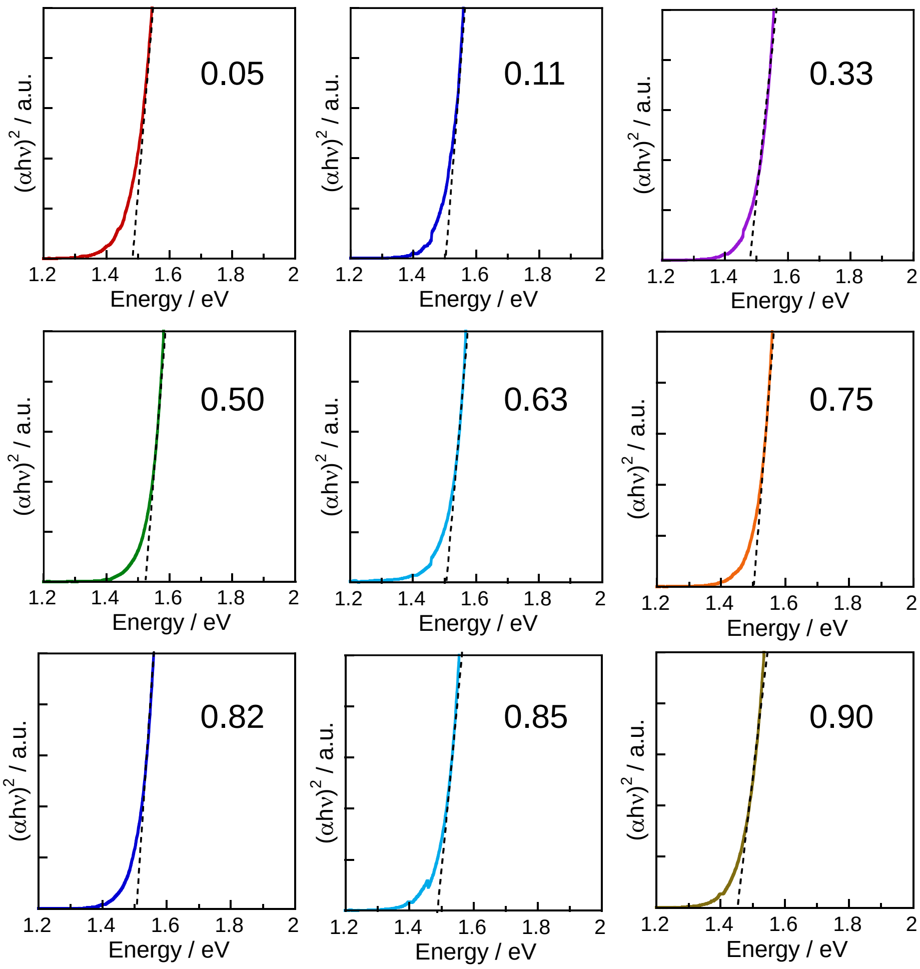

Figure S2. Plots of $(\alpha h\nu)^2$ as a function of photon energy (hν) (Tauc plots) for representative absorption spectra of Ag_8GeS_6 QDs prepared with various Ge/(Ag+Ge) ratios in the precursors.

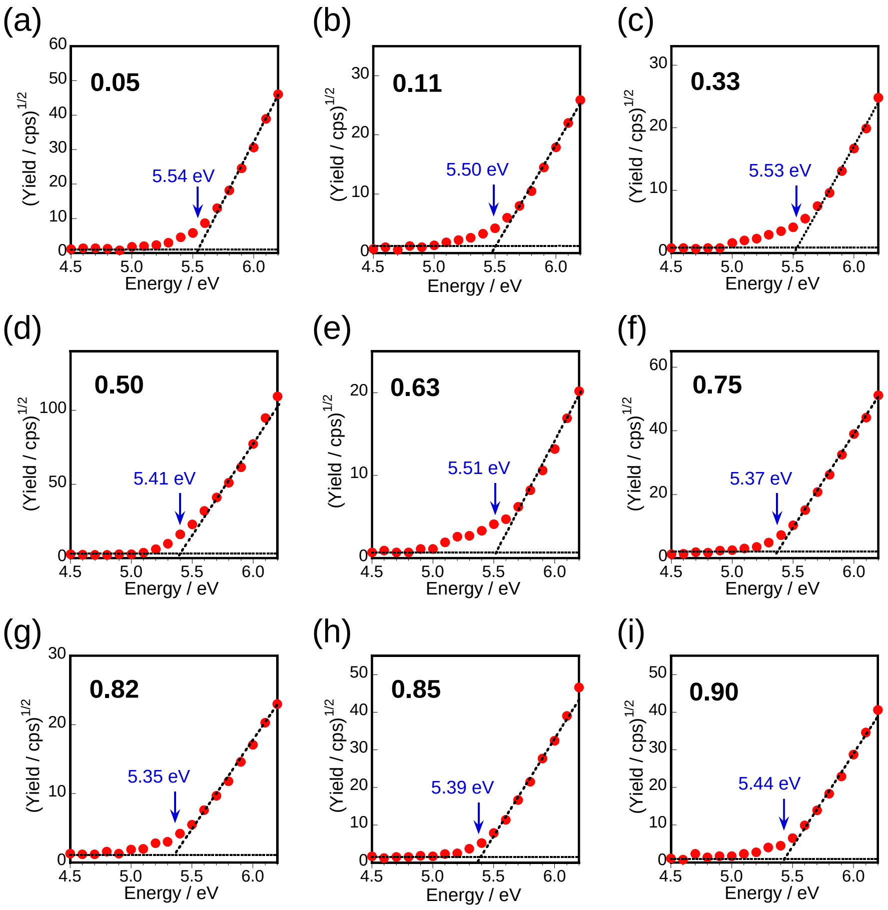

Figure S3. Representative photoelectron yield spectra of Ag_8GeS_6 QDs prepared with Ge/(Ag+Ge) = 0.05 (a), 0.11 (b), 0.33 (c), 0.50 (d), 0.63 (e), 0.75 (f), 0.82 (g), 0.85 (h), and 0.90 (i) in the precursors.

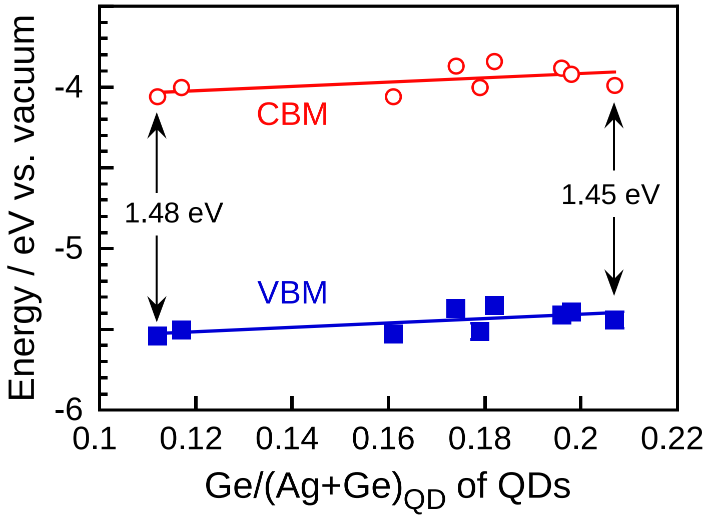

Figure S4. Energy levels of the valence band maximum (VBM) and the conduction band minimum (CBM) of Ag_8GeS_6 QDs as a function of their Ge/(Ag+Ge)$_{QD}$ ratio.

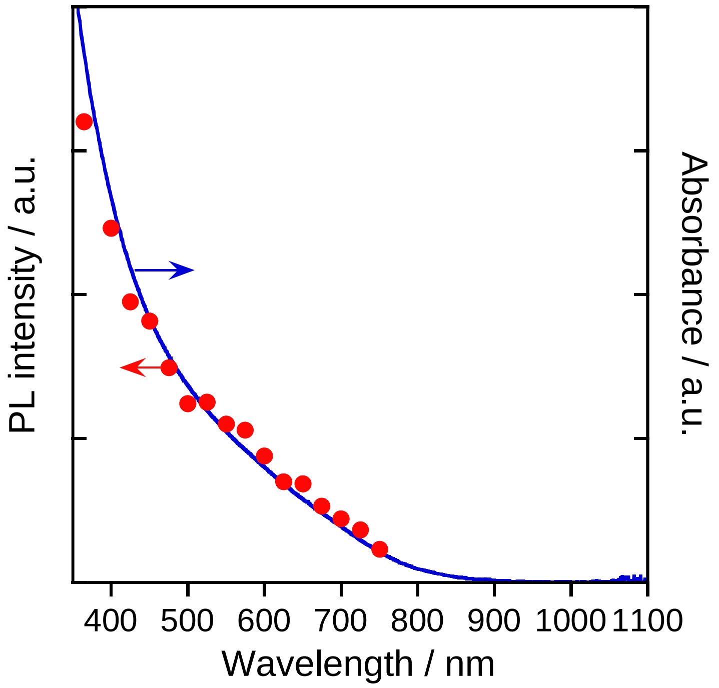

Figure S5. PL excitation spectrum (solid circles) of Ag_8GeS_6 QDs prepared with Ge/(Ag+Ge) = 0.82 in the precursors. The PL intensity was monitored at the wavelength of the PL peak. The corresponding absorption spectrum (solid line) is also shown.

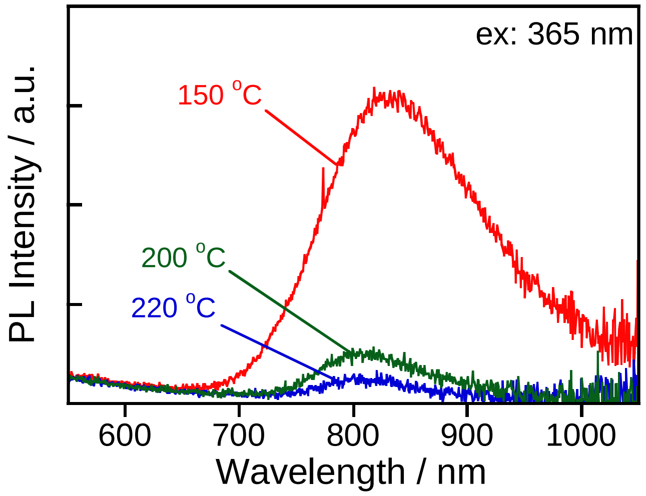

Figure S6. PL spectra of Ag_8GeS_6 QDs prepared at different heating temperatures. The Ge/(Ag+Ge) ratio in the precursors was 0.63. The wavelength of excitation light was 365 nm.

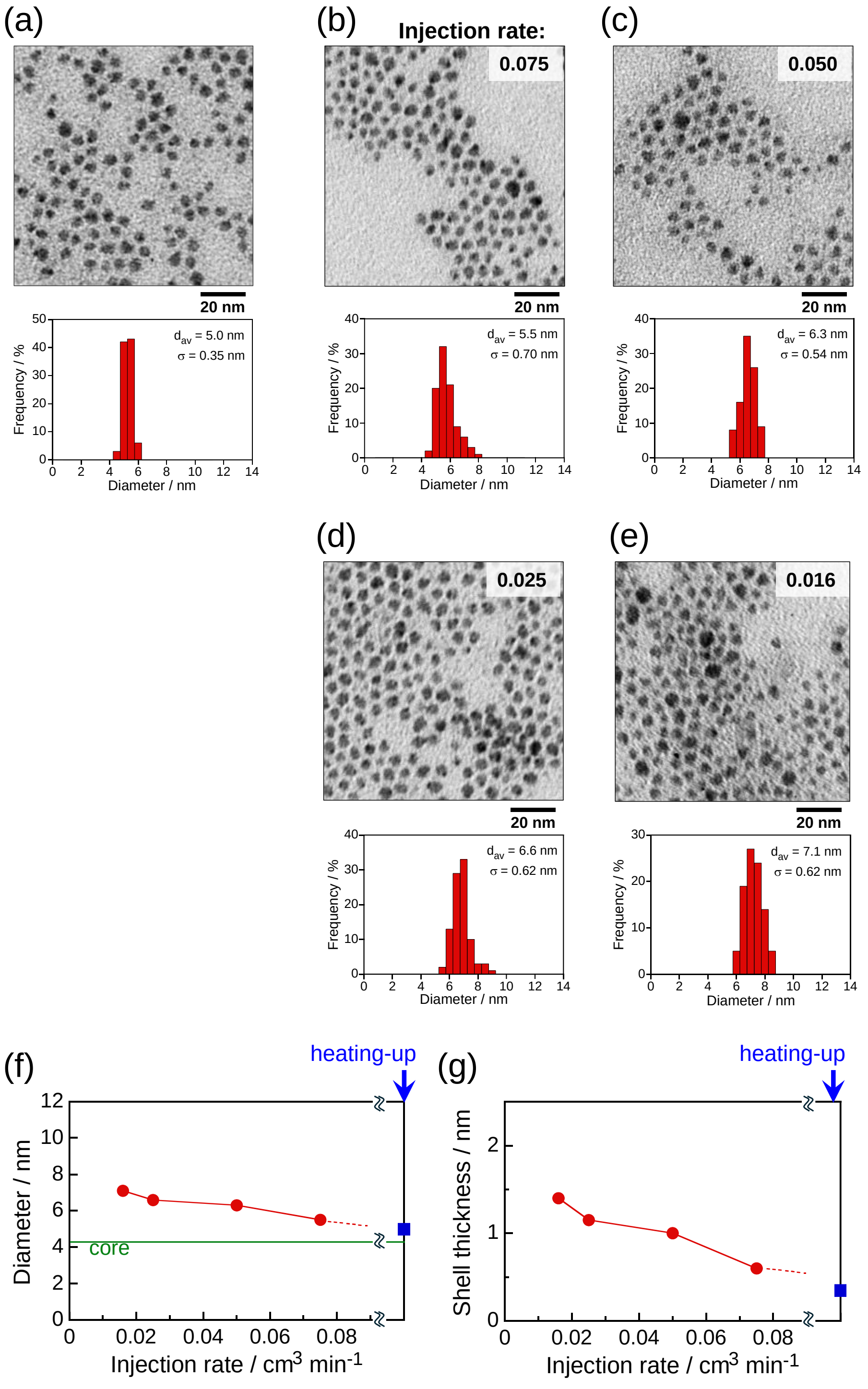

Figure S7. Wide-area TEM images and size distribution histograms of Ag_8GeS_6@ZnS core-shell QDs. The QDs were prepared by a heating-up method (a) or by the dropwise addition of a ZnS precursor solution with injection rates of 0.075 (b), 0.050 (c), 0.025 (d), and 0.016 (e) cm^3/min. (f,g) Changes in the average diameter (d_{av}) (f) and ZnS shell thickness (g) of Ag_8GeS_6@ZnS QDs depending on the preparation method.

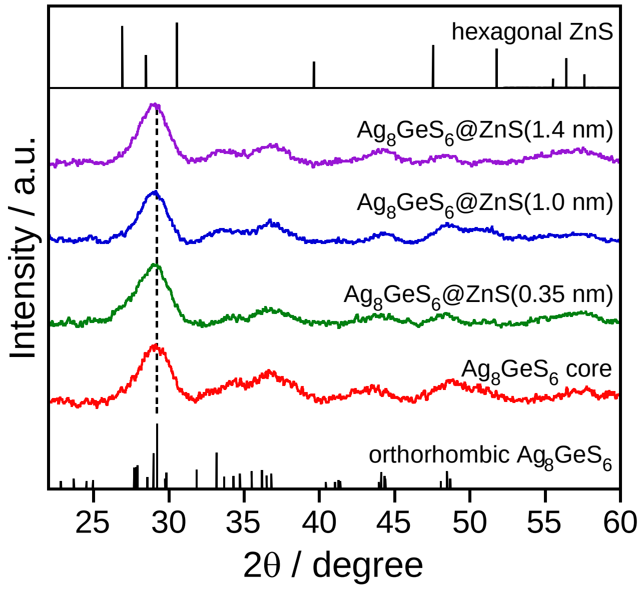

Figure S8. XRD patterns of Ag_8GeS_6 QDs and Ag_8GeS_6@ZnS QDs with different ZnS shell thicknesses. The Ag_8GeS_6 QDs used as a core were prepared with Ge/(Ag+Ge)= 0.82 in the precursors. The standard diffraction patterns of orthorhombic Ag_8GeS_6 (PDF card# 00-044-1416) and hexagonal ZnS (PDF card# 00-010-0434) are also shown as references.

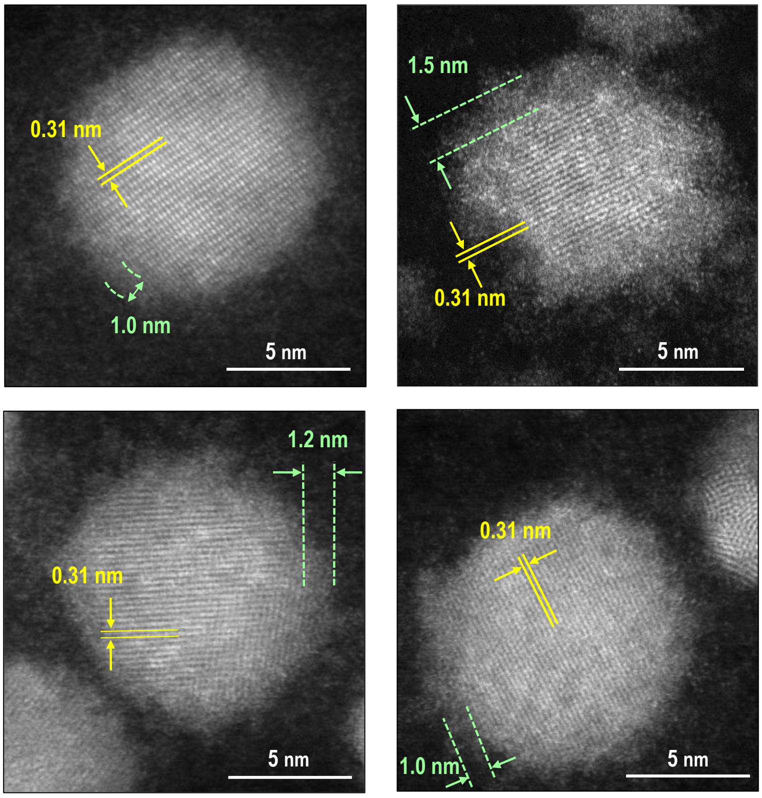

Figure S9. Representative HAADF-STEM images of Ag_8GeS_6@ZnS QDs with a ZnS shell thickness of 1.0 nm.

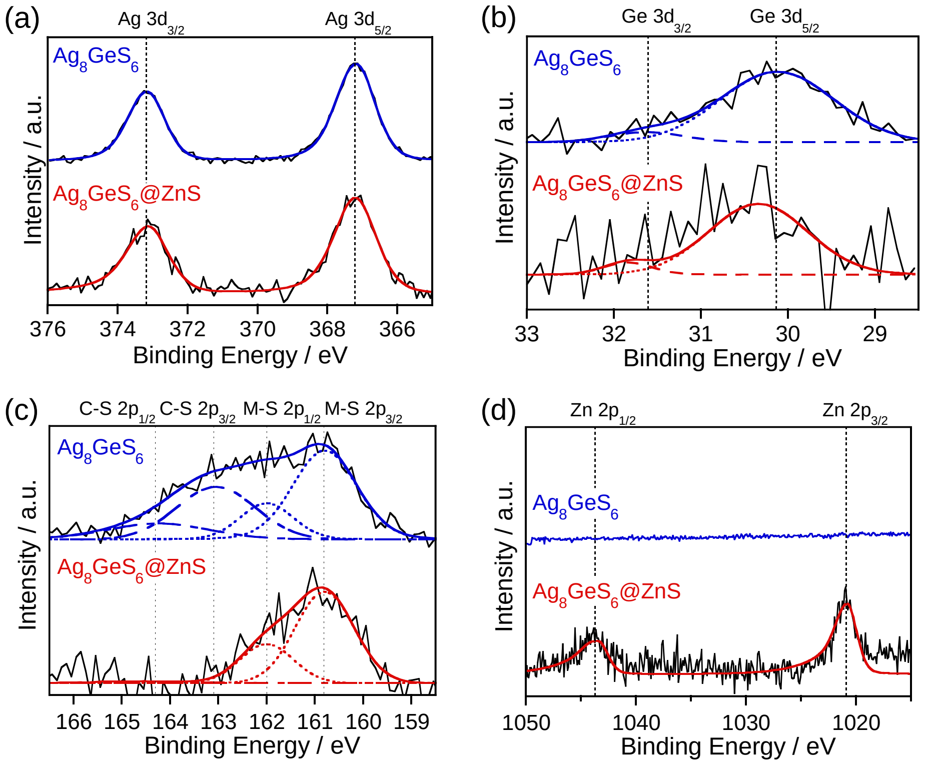

Figure S10. XPS spectra of Ag_8GeS_6 QDs and Ag_8GeS_6@ZnS QDs for (a) Ag 3d, (b) Ge 3d, (c) S 2p, and (d) Zn 2p.

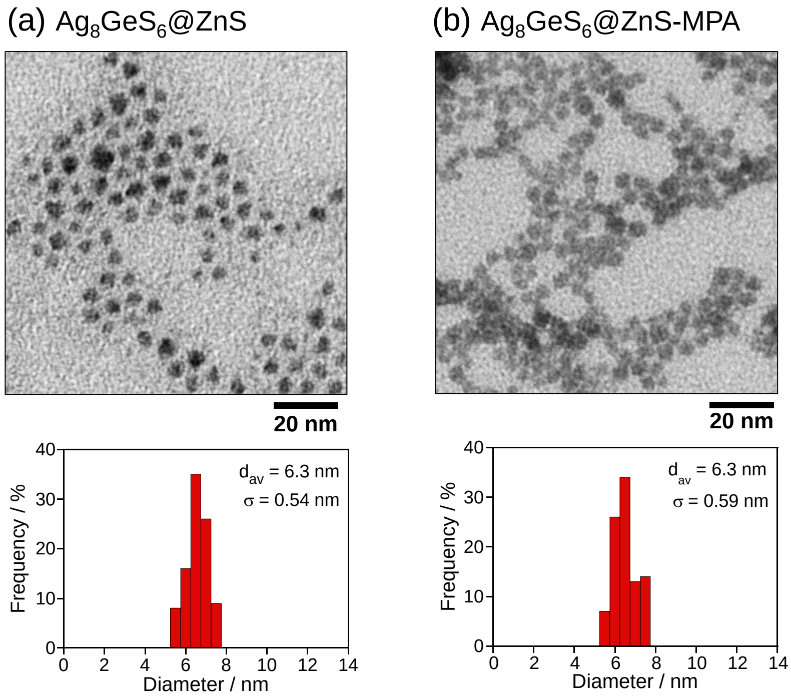

Figure S11. Wide-area TEM images and size distribution histograms of Ag_8GeS_6@ZnS QDs before (a) and after ligand exchange with MPA (b). The Ag_8GeS_6 QDs used as a core were prepared with Ge/(Ag+Ge)= 0.82 in the precursors. The thickness of the ZnS shell on QDs prepared by dropwise addition with an injection rate of 0.050 cm^3/min was 1.0 nm.

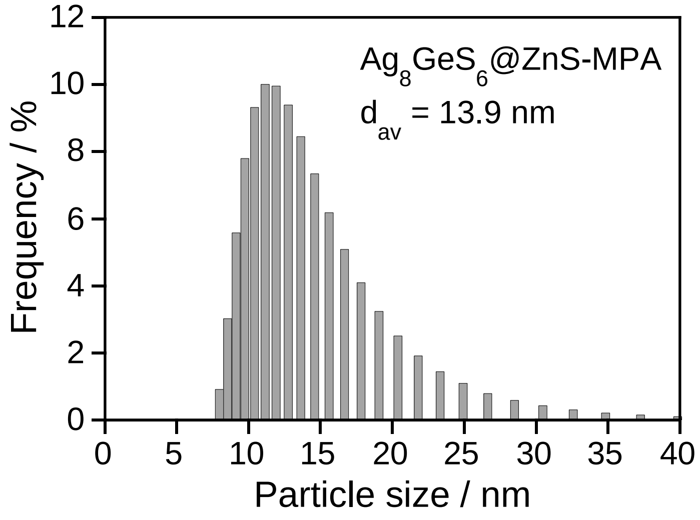

Figure S12. Size distribution of Ag_8GeS_6@ZnS-MPA dispersed in water, measured by DLS.

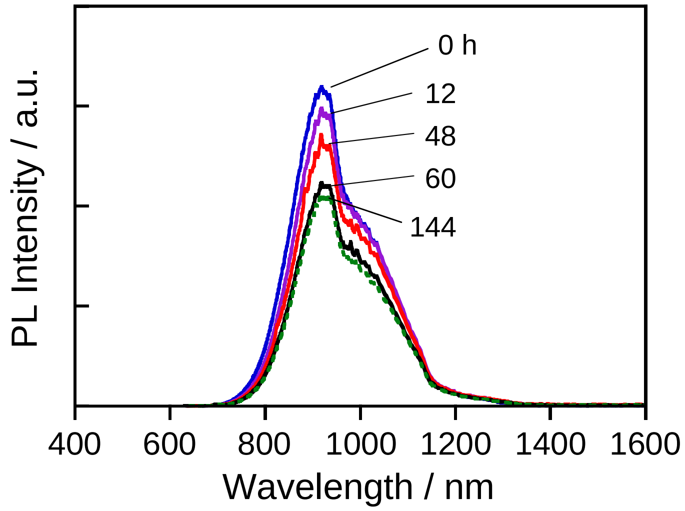

Figure S13. Changes in PL spectra of Ag_8GeS_6@ZnS-MPA QDs uniformly dispersed in water with irradiation of monochromatic light at 700 nm of 4.5 mW cm^{-2} in intensity at room temperature under an N_2 atmosphere. The Ag_8GeS_6 QDs used as a core were prepared with Ge/(Ag+Ge)= 0.82 in the precursors.

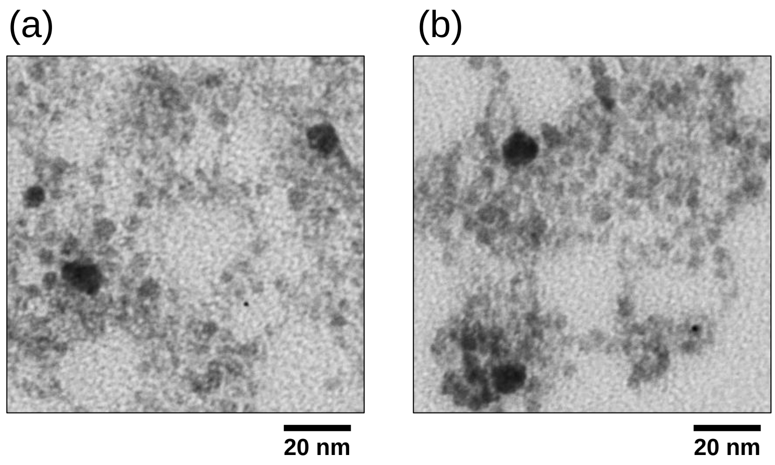

Figure S14. TEM images of Ag_8GeS_6@ZnS-MPA QDs subjected to (a) storage in the dark for 27 days and (b) irradiation by monochromatic light at 700 nm for 144 h.

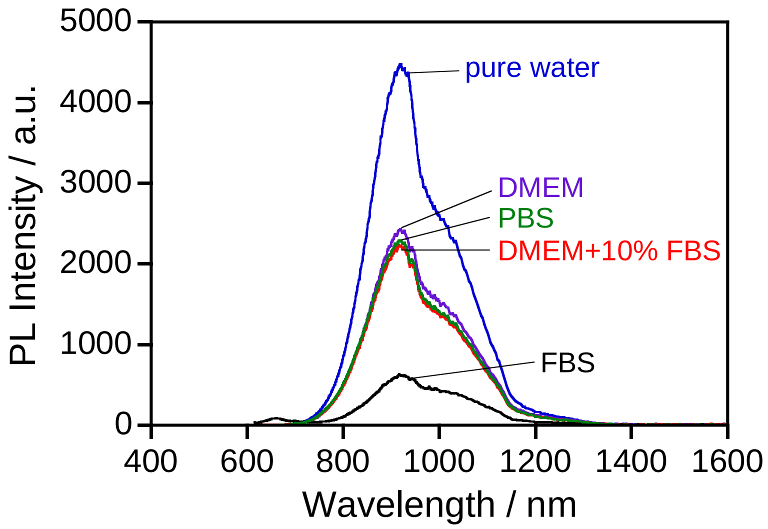

Figure S15. Initial PL spectra of Ag_8GeS_6@ZnS-MPA QDs in various types of biological media.

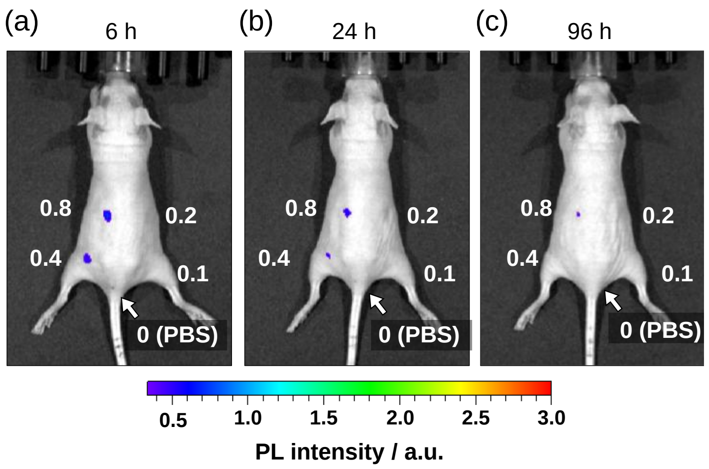

Figure S16. *In vivo* PL images of a mouse subcutaneously injected with Ag_8GeS_6@ZnS-MPA dispersions (each 50 mm^3) in the back at 6 h (a), 24 h (b), and 96 h (c) after injection. The numbers in the panels are the concentrations of QDs in the solutions injected in the unit of μmol(QDs) dm^{-3}.

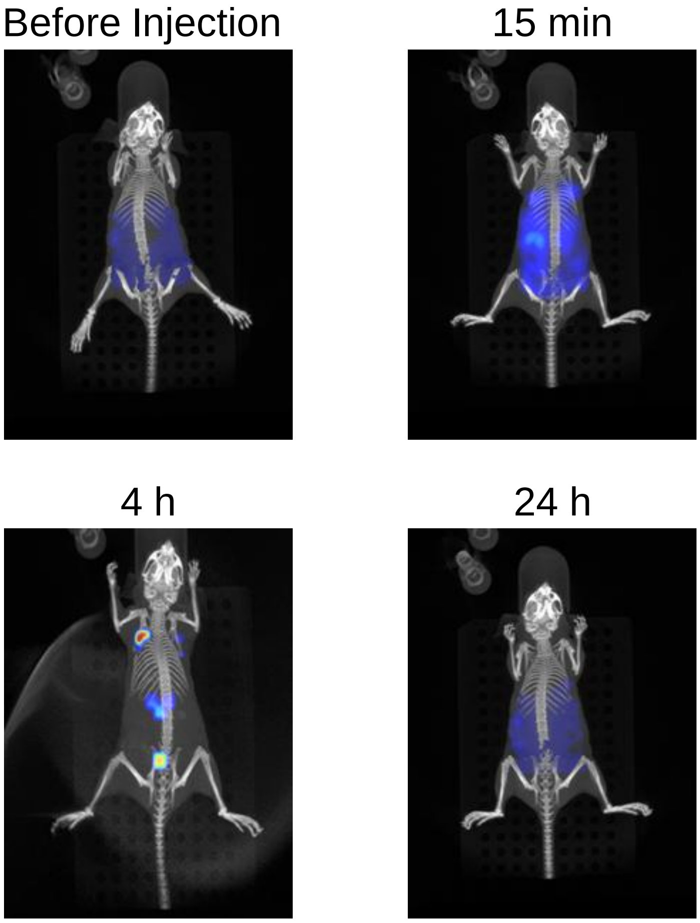

Figure S17. Three-dimensional PL images at various time points after injection. Each PL image was overlaid onto the corresponding X-ray CT image of the mouse under intravenous administration of Ag_8GeS_6@ZnS-MPA QDs. The PL images were measured at the times indicated above the panels.

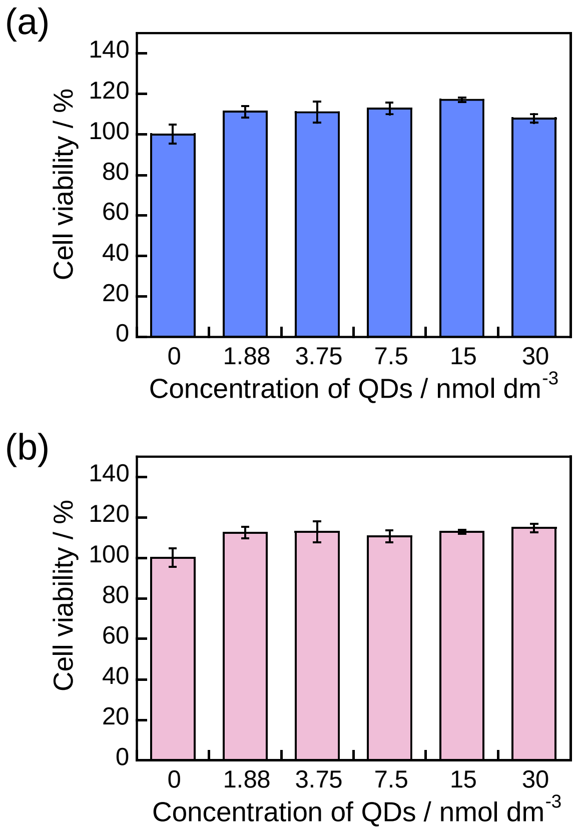

Figure S18. Cell viability of (a) HeLa cells and (b) Swiss 3T3 cells after incubation with Ag_8GeS_6@ZnS-MPA. The data, each in triplicate, are shown as the mean ± SD values. The significance of individual differences was evaluated using Student's t-test.

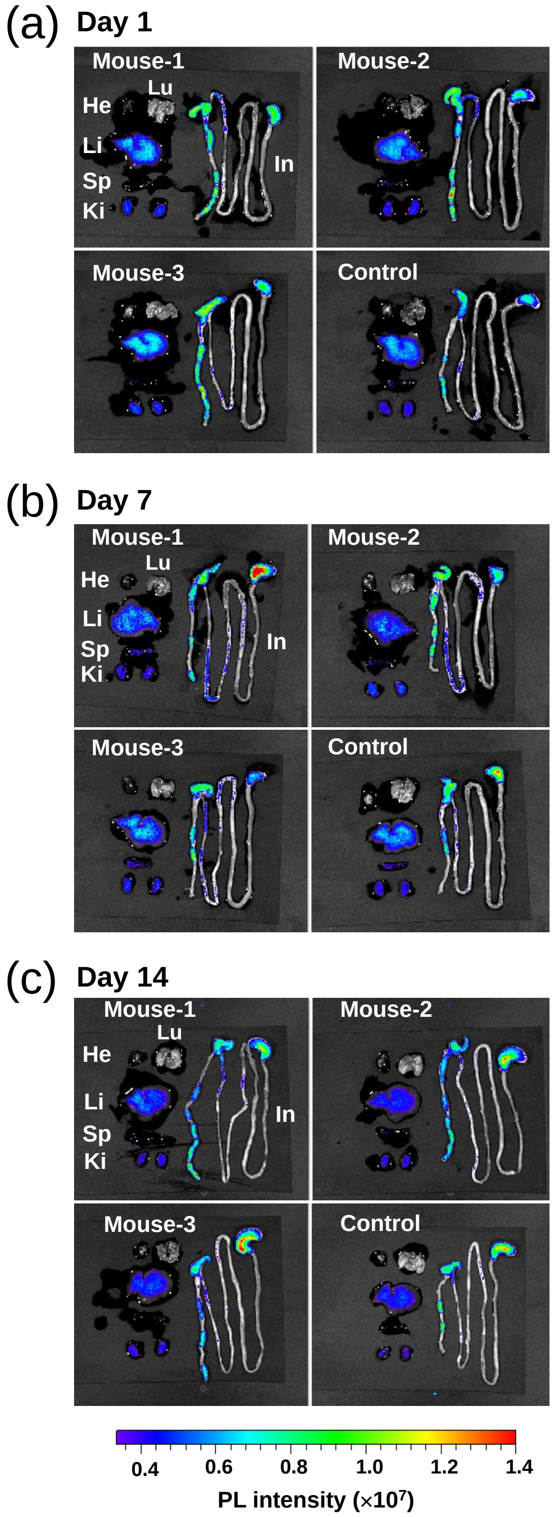

Figure S19. Photoluminescence distribution in major organs of mice injected with Ag_8GeS_6@ZnS-MPA QDs on day 1 (a), day 7 (b), and day 14 (c). PL signals were detected with an *in vivo* PL imaging system (IVIS Spectrum CT; excitation filter: 640 nm; emission filter: 840 nm). In: intestine; He: heart; Lu: lungs; Li: liver; Sp: spleen; Ki: kidneys.
Mice were divided into two groups: an Ag_8GeS_6@ZnS-MPA QD group (three mice: mouse 1, mouse 2, and mouse 3) and a PBS control group (one mouse (control)). Each mouse received a 0.10 cm^3 subcutaneous injection.

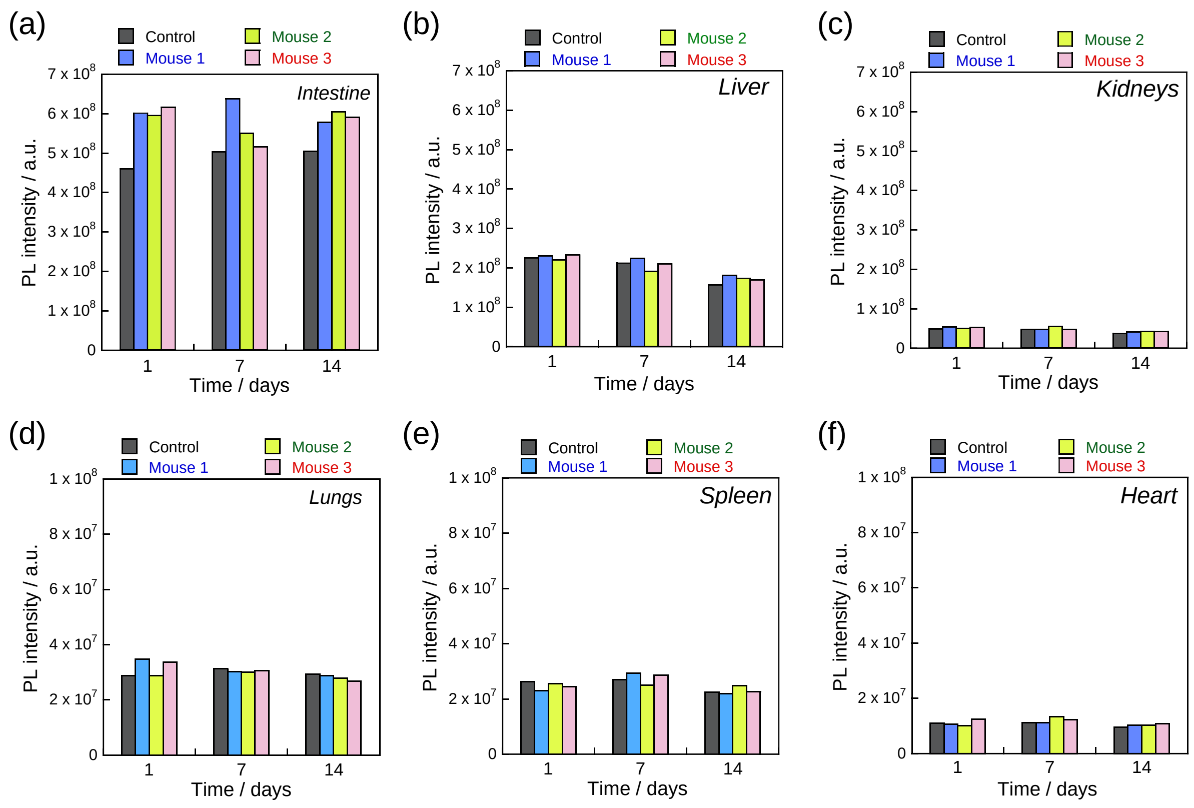

Figure S20. Changes in the average PL intensities of major organs over time after Ag_8GeS_6@ZnS-MPA QD injection: (a) intestine, (b) liver, (c) kidneys, (d) lungs, (e) spleen, and (f) heart.

Mice were divided into two groups: an Ag_8GeS_6@ZnS-MPA QD group (three mice: mouse 1, mouse 2, and mouse 3) and a PBS control group (one mouse (control)). Each mouse received a 0.10 cm^3 subcutaneous injection.

Table S1. Chemical compositions of Ag_8GeS_6 QDs prepared with various Ge/(Ag+Ge) ratios in the precursors.

Ge/(Ag+Ge) in precursors	Composition (%) Ag	Ge	S	$Ge/(Ag+Ge)_{QD}$ in QDs
0.05	55.5	7.0	37.5	0.11
0.11	54.1	7.2	38.7	0.12
0.33	49.9	9.6	40.5	0.16
0.50	47.7	11.7	40.7	0.20
0.63	47.9	10.5	41.6	0.18
0.75	49.2	10.4	40.4	0.17
0.82	48.3	10.8	40.9	0.18
0.85	48.4	12.0	39.6	0.20
0.90	47.4	12.4	40.2	0.21

Table S2. Chemical compositions of Ag_8GeS_6, Ag_8GeS_6@ZnS, and Ag_8GeS_6@ZnS-MPA QDs evaluated by ensemble measurements with XPS and EDS.

Sample	Method	Composition (%) Ag	Ge	S	Zn	$Ge/(Ag+Ge)_{QD}$
Ag_8GeS_6	EDS	48.3	10.8	40.9	0.0	0.18
	XPS	40.0	11.1	48.9	0.0	0.19
Ag_8GeS_6@ZnS	EDS	30.8	9.0	40.7	19.5	0.23
	XPS	17.4	3.4	31.6	47.6	0.16
Ag_8GeS_6@ZnS-MPA	EDS	20.8	4.3	53.5	21.4	0.17

The Ag_8GeS_6 QDs used as a core were prepared with Ge/(Ag+Ge)= 0.82 in the precursors. The thickness of the ZnS shell was 1.0 nm.

Table S3. Characteristics of low-toxicity Ag-based QDs exhibiting NIR PL, as reported in previous studies, for comparison with the QDs in this study.

QDs	Averagae particle size (nm)	PL peak wavelength (nm)	PL QY in organic solvent (%)	PL QY in aqueous solution (%)	PL lifetime (ns)	Application	Ref.
Ag_2S	2.1	653	-	1.2	-	*In vitro* imaging	[1]
Ag_2S	2.5	978	-	1.3	-	*In vivo*, *in vitro* imaging	[2]
Ag_2S	9	1220	5.1	1.6	~630	*In vivo* imaging	[3]
Ag_2S	12	1200	2.3	2.3	~1200	*In vivo* imaging	[4]
Ag_2S	9.8	1200	10	10	~3800	*In vivo* imaging	[5]
Ag_2S	5.4	1058	-	15.5	-	*In vivo* imaging	[6]
Ag_2S	6.1	820	-	14.1	-	*In vivo* imaging	[7]
Ag_2S	5.4	1200	-	15.5	-	*In vivo* imaging	[8]
Ag_2Se	2.4	820	-	3.09	-	*In vivo* imaging	[9]
Ag_2Se	2.4	750	-	11.2	-	*In vivo* imaging	[10]
Ag_2Se	16.4	930	10.2	7.3	-	*In vivo* imaging	[11]
Ag_2Se	3.5	1300	-	19.4	-	*In vivo* imaging	[12]
Ag_2Te	3.4	1000-1700	~6.51	~4.94	-	*In vivo* imaging	[13]
Ag_2Te	9.5	1085	~20.5	22.8	-	*In vitro* imaging	[14]
Ag_2Te	4.7	1068	-	15.2	-	*In vitro* imaging	[15]
$Ag_2Te@Ag_2Se$	5.4	1732	0.21	0.21	12.72	*In vivo* imaging	[16]

Table S3. (continued)

QDs	Averagae particle size (nm)	PL peak wavelength (nm)	PL QY in organic solvent (%)	PL QY in aqueous solution (%)	PL lifetime (ns)	Application	Ref.
$AgInS_2$	5.2	617	-	20.3	~194.9	*In vitro* imaging	[17]
$AgInS_2$	3.5	800	-	35	~870	*In vivo* imaging	[18]
$AgInS_2$	6.3	817	-	34.4	853.1	*In vivo* imaging	[19]
$AgInS_2$@ZnS	2.5	814	1.5	1.5	394.4	-	[20]
$AgInS_2$@ZnS	~12	740	~28	~10	-	*In vivo* imaging	[21]
$AgInSe_2$@ZnSe	3.2	675	-	5.6	-	*In vitro* imaging	[22]
AgInGaSe@ GaS	5.2	800	38	6.2	~220	*In vivo* imaging	[23]
$AgInTe_2$	length: 11.4 width: 5.5	1033	18	2.2	-	*In vivo* imaging	[24]
Ag–Au–Se	4.37	978	65.3	-	4580	-	[25]
**Ag_8GeS_6@ ZnS**	**6.3**	**900**	**40-42**	**23**	**486**	***In vivo* imaging**	**This work**

Table S4. PL lifetime components of Ag_8GeS_6 and Ag_8GeS_6@ZnS (ZnS shell thickness: 1.0 nm) QDs.

Sample	<τ> (ns)	τ_1 (ns)	A_1 (%)	τ_2 (ns)	A_2 (%)	τ_3 (ns)	A_3 (%)	χ^2	PLQY (%)	k_{nr} ($\times10^6$ s^{-1})	k_{rad} ($\times10^6$ s^{-1})
Ag_8GeS_6	213	43	43	123	44	377	13	1.0	11	4.2	0.52
Ag_8GeS_6@ZnS	486	33	42	213	36	669	22	1.0	40	1.2	0.82

The Ag_8GeS_6 QDs used as a core were prepared with Ge/(Ag+Ge)= 0.82 in the precursors.

Table S5. Results of biochemical analysis of serum from mice administered Ag_8GeS_6@ZnS-MPA QDs.

Time (days)	Sample	ALB (g/dL)	AST (IU/L)	ALT (IU/L)	LDH (IU/L)	γ-GT (IU/L)	T-BIL (mg/dL)
1	Control	3.3	66	14	274	< 3	0.08
	Mouse 1	3.3	53	19	433	< 3	0.14
	Mouse 2	3.4	47	18	234	< 3	0.14
	Mouse 3	3.2	44	13	161	< 3	0.13
7	Control	3.6	94	18	619	< 3	0.07
	Mouse 1	3.5	84	25	640	< 3	0.12
	Mouse 2	3.5	105	30	364	< 3	0.09
	Mouse 3	3.5	86	26	483	< 3	0.11
14	Control	3.3	68	15	456	< 3	0.14
	Mouse 1	3.5	100	22	465	< 3	0.11
	Mouse 2	3.3	74	26	238	< 3	0.11
	Mouse 3	3.3	71	21	282	< 3	0.07

Mice were divided into two groups: an Ag_8GeS_6@ZnS-MPA QD group (three mice: mouse 1, mouse 2, and mouse 3) and a PBS control group (one mouse (control)). Each mouse received a 0.10 cm^3 subcutaneous injection.
Markers are albumin (ALB), aspartate aminotransferase (AST), alanine aminotransferase (ALT), lactate dehydrogenase (LDH), γ-glutamyl transferase (γ-GT), and total bilirubin (T-BIL), which are commonly associated with liver irritation and potential liver stress due to QD accumulation.

References

[1] C. Wang, Y. Wang, L. Xu, et al., Facile Aqueous-Phase Synthesis of Biocompatible and Fluorescent Ag_2S Nanoclusters for Bioimaging: Tunable Photoluminescence from Red to Near Infrared, *Small* **2012**, *8*, 3137.

[2] J. Gao, C. Wu, D. Deng, P. Wu, C. Cai, Direct Synthesis of Water-Soluble Aptamer-Ag_2S Quantum Dots at Ambient Temperature for Specific Imaging and Photothermal Therapy of Cancer, *Adv. Healthc. Mater.* **2016**, *5*, 2437.

[3] J. W. de Wit, I. Zabala-Gutierrez, R. Marin, et al., New Insights in Luminescence and Quenching Mechanisms of Ag_2S Nanocrystals through Temperature-Dependent Spectroscopy, *J. Phys. Chem. Lett.* **2024**, *15*, 8420.

[4] A. Ortega-Rodríguez, Y. Shen, I. Zabala Gutierrez, et al., 10-Fold Quantum Yield Improvement of Ag_2S Nanoparticles by Fine Compositional Tuning, *ACS Appl. Mater. Interfaces* **2020**, *12*, 12500.

[5] I. Z. Gutierrez, C. Gerke, Y. Shen, et al., Boosting the Near-Infrared Emission of Ag_2S Nanoparticles by a Controllable Surface Treatment for Bioimaging Applications, *ACS Appl. Mater. Interfaces* **2022**, *14*, 4871.

[6] Y. Zhang, G. Hong, Y. Zhang, et al., Ag_2S Quantum Dot: A Bright and Biocompatible Fluorescent Nanoprobe in the Second Near-Infrared Window, *ACS Nano* **2012**, *6*, 3695.

[7] R. Tang, J. Xue, B. Xu, D. Shen, G. P. Sudlow, S. Achilefu, Tunable Ultrasmall Visible-to-Extended Near-Infrared Emitting Silver Sulfide Quantum Dots for Integrin-Targeted Cancer Imaging, *ACS Nano* **2015**, *9*, 220.

[8] G. Hong, J. T. Robinson, Y. Zhang, et al., *In Vivo* Fluorescence Imaging with Ag_2S Quantum Dots in the Second Near-Infrared Region, *Angew. Chem. Int. Ed.* **2012**, *51*, 9818.

[9] Y.-P. Gu, R. Cui, Z.-L. Zhang, Z.-X. Xie, D.-W. Pang, Ultrasmall Near-Infrared Ag_2Se Quantum Dots with Tunable Fluorescence for *In Vivo* Imaging, *J. Am. Chem. Soc.* **2012**, *134*, 79.

[10] X.-L. Ge, B. Huang, Z.-L. Zhang, et al., Glucose-Functionalized Near-Infrared Ag_2Se Quantum Dots with Renal Excretion Ability for Long-Term *In Vivo* Tumor Imaging, *J. Mater. Chem. B* **2019**, *7*, 5782.

[11] C.-N. Zhu, G. Chen, Z.-Q. Tian, et al., Near-Infrared Fluorescent Ag_2Se–Cetuximab Nanoprobes for Targeted Imaging and Therapy of Cancer, *Small* **2017**, *13*, 1602309.

[12] B. Dong, C. Li, G. Chen, et al., Facile Synthesis of Highly Photoluminescent Ag_2Se Quantum Dots as a New Fluorescent Probe in the Second Near-Infrared Window for *In Vivo* Imaging, *Chem. Mater.* **2013**, *25*, 2503.

[13] X.-H. Shi, Y.-Y. Dai, L. Wang, Z.-G. Wang, S.-L. Liu, Water-Soluble High-Quality Ag_2Te Quantum Dots Prepared by Mutual Adaptation of Synthesis and Surface Modification for *In Vivo* Imaging, *ACS Applied Bio Materials* **2021**, *4*, 7692.

[14] H. Jin, R. Gui, J. Sun, Y. Wang, Glycerol-Regulated Facile Synthesis and Targeted Cell Imaging of Highly Luminescent Ag_2Te Quantum Dots with Tunable Near-Infrared Emission, *Colloids Surf. B Biointerfaces* **2016**, *143*, 118.

[15] M. Yang, R. Gui, H. Jin, et al., Ag_2Te Quantum Dots with Compact Surface Coatings of Multivalent Polymers: Ambient One-Pot Aqueous Synthesis and the Second Near-Infrared Bioimaging, *Colloids Surf. B Biointerfaces.* **2015**, *126*, 115.

[16] K. Wang, K.-H. Deng, Y.-S. Tian, et al., Core/Shell-Structured Ag_2Te/Ag_2Se Quantum Dots for High-Resolution *In Vivo* Fluorescence Imaging in the Near Infrared IIb Region, *ACS Appl. Nano Mater.* **2023**, *6*, 14289.

[17] M. Hashemkhani, M. Loizidou, A. J. MacRobert, H. Yagci Acar, One-Step Aqueous Synthesis of Anionic and Cationic $AgInS_2$ Quantum Dots and Their Utility in Improving the Efficacy of ALA-Based Photodynamic Therapy, *Inorg. Chem.* **2022**, *61*, 2846.

[18] L. Liu, I. Hu R Fau - Roy, G. Roy I Fau - Lin, et al., Synthesis of Luminescent Near-Infrared $AgInS_2$ Nanocrystals as Optical Probes for *In Vivo* Applications, *Theranostics* **2013**, *3*, 109.
[19] L. Tan, S. Liu, X. Li, I. S. Chronakis, Y. Shen, A New Strategy for Synthesizing $AgInS_2$ Quantum Dots Emitting Brightly in Near-Infrared Window for *In Vivo* Imaging, *Colloids Surf. B Biointerfaces* **2015**, *125*, 222.
[20] B. Mao, C.-H. Chuang, C. McCleese, J. Zhu, C. Burda, Near-Infrared Emitting $AgInS_2$/ZnS Nanocrystals, *J. Phys. Chem. C* **2014**, *118*, 13883.
[21] A. Shamirian, O. Appelbe, Q. Zhang, B. Ganesh, S. J. Kron, P. T. Snee, A Toolkit for Bioimaging Using Near-Infrared $AgInS_2$/ZnS Quantum Dots, *J. Mater. Chem. B* **2015**, *3*, 8188.
[22] O. S. Oluwafemi, B. M. M. May, S. Parani, N. Tsolekile, Facile, Large Scale Synthesis of Water Soluble $AgInSe_2$/ZnSe Quantum Dots and Its Cell Viability Assessment on Different Cell Lines, *Mater. Sci. Eng. C* **2020**, *106*, 110181.
[23] T. Kameyama, H. Yamauchi, T. Yamamoto, et al., Tailored Photoluminescence Properties of Ag(In,Ga)Se_2 Quantum Dots for Near-Infrared *In Vivo* Imaging, *ACS Appl. Nano Mater.* **2020**, *3*, 3275.
[24] T. Kameyama, Y. Ishigami, H. Yukawa, et al., Crystal Phase-Controlled Synthesis of Rod-Shaped $AgInTe_2$ Nanocrystals for *In Vivo* Imaging in the Near-Infrared Wavelength Region, *Nanoscale* **2016**, *8*, 5435.
[25] H. Yang, R. Li, Y. Zhang, et al., Colloidal Alloyed Quantum Dots with Enhanced Photoluminescence Quantum Yield in the NIR-II Window, *J. Am. Chem. Soc.* **2021**, *143*, 2601.
